# Plasma Catalysis Modeling: How Ideal Is Atomic Hydrogen for Eley–Rideal?

**DOI:** 10.1021/acs.jpcc.4c02193

**Published:** 2024-07-01

**Authors:** Roel Michiels, Nick Gerrits, Erik Neyts, Annemie Bogaerts

**Affiliations:** † Research group PLASMANT, Department of Chemistry, University of Antwerp, Universiteitsplein 1, Wilrijk,Antwerp BE-2610, Belgium; ‡ Leiden Institute of Chemistry, Gorlaeus Laboratories, Leiden University, P.O. Box 9502, Leiden 2300 RA, The Netherlands

## Abstract

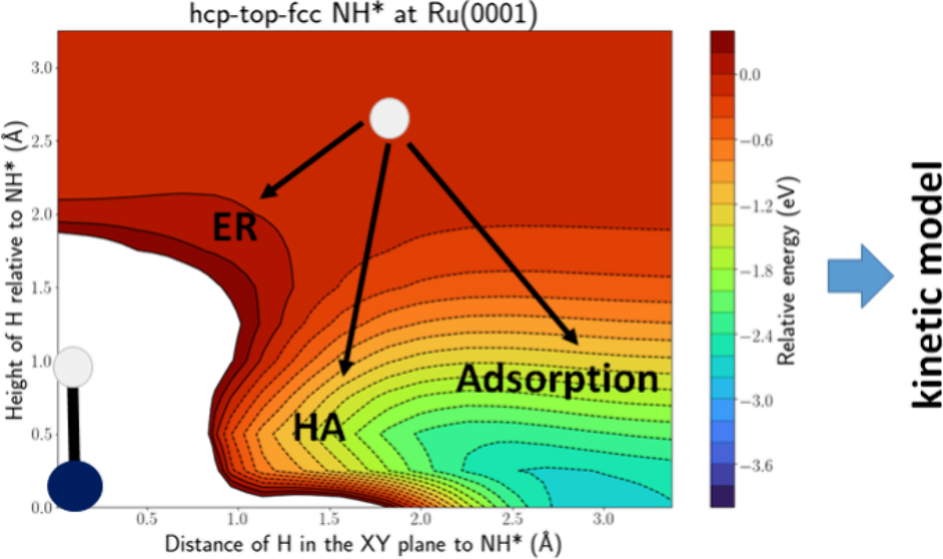

Plasma catalysis is an emerging technology, but a lot of questions about the underlying surface mechanisms remain unanswered. One of these questions is how important Eley–Rideal (ER) reactions are, next to Langmuir–Hinshelwood reactions. Most plasma catalysis kinetic models predict ER reactions to be important and sometimes even vital for the surface chemistry. In this work, we take a critical look at how ER reactions involving H radicals are incorporated in kinetic models describing CO_2_ hydrogenation and NH_3_ synthesis. To this end, we construct potential energy surface (PES) intersections, similar to elbow plots constructed for dissociative chemisorption. The results of the PES intersections are in agreement with *ab initio* molecular dynamics (AIMD) findings in literature while being computationally much cheaper. We find that, for the reactions studied here, adsorption is more probable than a reaction via the hot atom (HA) mechanism, which in turn is more probable than a reaction via the ER mechanism. We also conclude that kinetic models of plasma-catalytic systems tend to overestimate the importance of ER reactions. Furthermore, as opposed to what is often assumed in kinetic models, the choice of catalyst will influence the ER reaction probability. Overall, the description of ER reactions is too much “ideal” in models. Based on our findings, we make a number of recommendations on how to incorporate ER reactions in kinetic models to avoid overestimation of their importance.

## 1 Introduction

Plasma catalysis is an emerging technology for greenhouse gas conversion into value-added products. It could contribute to the transition from a fossil fuel-based chemical industry to an electrified chemical industry. Indeed, plasma is powered by electricity and can easily be switched on and off. Hence, it allows the conversion of reactants using fluctuating renewable energy sources, rather than thermal energy, often generated by the burning of fossil fuels, used in conventional catalysis. Due to the high reactivity of a plasma and the selectivity of a catalyst, plasma catalysis might be particularly useful for the conversion of hard-to-activate molecules, like CO_2_ and N_2_, into value-added chemicals like CH_3_OH and NH_3_. Thus, plasma catalysis could also be used to reduce CO_2_ emissions by converting CO_2_ instead of emitting it into the atmosphere. This can help reduce the acceleration of climate change.^1,2^

Both the plasma-catalytic conversion of CO_2_ into value-added chemicals, like CH_3_OH or CH_4_, as well as NH_3_ synthesis have attracted considerable interest in recent years. Despite this growing interest, the underlying mechanisms are not yet fully understood. Plasma catalysis is a complex process, because a plasma and catalyst can affect each other in various ways. These effects can be chemical in nature, e.g., the impact of plasma-generated radicals and excited species on the surface chemistry, or physical, e.g., the modification of the electric field by the catalyst. In some cases, these interactions between a plasma and catalyst can cause a synergistic effect, i.e., the combined effect of plasma catalysis is larger than the sum of plasma alone and catalysis alone. However, it is important to note that this synergistic effect is not universal, as it only appears under certain circumstances and for some systems. The interactions between a plasma and catalyst can also affect the product selectivity. This can be interesting to tune the conversion toward the more desired products.^2−5^

To better understand the underlying mechanisms in plasma catalysis, further research is needed. More experimental studies are much needed, but they have the inherent disadvantage that they cannot, or only to a limited extent, disentangle all possible underlying mechanisms. Computer simulations, on the other hand, allow us to study the various mechanisms separately. For plasma catalysis, different levels of modeling are needed, ranging from atomic scale models, like molecular dynamics (MD) and density functional theory (DFT) calculations, over kinetic models to fluid dynamics reactor models.^2,3^

**Figure 1:**
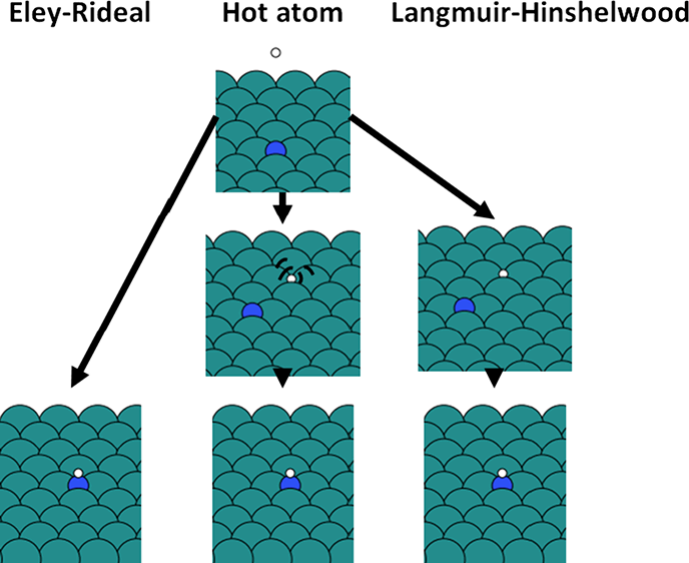
Overview of the three possible reaction mechanisms at a metal surface.

Of particular interest in plasma catalysis are the surface reaction mechanisms. Next to Langmuir–Hinshelwood (LH) reactions, common in thermal catalysis, Eley–Rideal (ER) reactions of plasma-generated radicals have been suggested to occur in plasma catalysis.^3−5^ In the ER mechanism, a gaseous species reacts with an adsorbed species through a direct collision, e.g., a gas-phase H atom reacts with an adsorbed N atom. As the gaseous species does not thermalize with the surface, the ER mechanism can be viewed as the limiting case of a nonthermal surface reaction. In contrast, in the LH mechanism, two thermalized adsorbed species react with each other, e.g., adsorbed H reacts with adsorbed N. Opposite to the ER mechanism, the LH mechanism can be seen as the limiting case of a thermal surface reaction. In between these two limiting cases, we can distinguish a third possible mechanism, i.e., the hot atom (HA) mechanism, where the impinging gas species does not directly collide with the surface species, but diffuses over the surface without thermalizing fully, before reacting with the adsorbed surface species. These three mechanisms are depicted in Figure 1.^6,7^

It is worth noting that the ER mechanism described above should actually be called Langmuir–Rideal, as Langmuir was the first to describe this mechanism. Originally, the ER mechanism was described as a reaction between a thermalized physisorbed and thermalized chemisorbed species.^8^ However, for the sake of clarity, we will stick to the terminology as explained in the previous paragraph, as this terminology is most often used in literature.^3,6,7^

The aim of our paper is to critically investigate the importance attributed to ER reactions by plasma catalysis models. Therefore, we will first discuss in Section 2 how ER reactions are modeled in kinetic models for plasma catalysis, as well as more fundamental modeling studies of ER reactions, highlighting any discrepancies with the kinetic models. Section 2 will also clearly motivate the aim of our work, which will be explained further in Section 3. In Section 4, we describe the methodology, while the results will be discussed in Section 5.

## 2 Modeling of Eley–Rideal Reactions

### 2.1 Kinetic Models for Plasma Catalysis

Several kinetic modeling studies of plasma-catalytic systems include ER reactions in their reaction set and conclude, to a varying extent, that these ER reactions can shift the selectivity toward more desired products or lower the overall reaction barrier. We will discuss some of these models here, focusing on studies that investigated N/H chemistry, i.e., NH_3_ synthesis, and C/O/H chemistry, i.e., CO_2_ hydrogenation and CH_4_ reforming. In the rest of this paper, we will denote adsorbed species with *, e.g., H*, and gas-phase species with (g), e.g., H(g). We will limit ourselves to low-temperature plasmas and, therefore, “low” translational energies. That is, we consider a translational energy regime considerably lower than the regime where typical binary collision models, based mainly on mass ratios, perform reasonably. This in turn makes the shape of the potential energy surface extremely important for the reaction dynamics.

Carrasco et al.^9^ modeled N_2_/H_2_ plasmas and compared their findings to experiments. They included ER reactions of the type NH_*x*_(g) + H* → NH_*x*+1_*, and H(g) + NH_x_* → NH_*x*+1_*, and found that inclusion of these ER reactions was necessary to explain the neutral densities measured in the experiments. The rate of ER reactions was calculated in the same way as adsorption rates and depended on several reactor-specific parameters and an ER sticking coefficient. The value of this sticking coefficient was chosen to obtain an optimal global agreement with experimental data, but it did not discriminate between the different adsorbates, e.g., the ER reactions of H(g) with N*, NH*, and NH_2_* had the same sticking coefficients. The approach and ER reactions of Carrasco et al.^9^ were subsequently adopted by Hong et al.,^10^ Jimenez-Redondo et al.,^11^ van ‘t Veer et al.^12^ and Chen et al.^13^ They all concluded that ER reactions contribute significantly to the production of NH_3_. Similar conclusions were reached by Shao and Mesbah^14^ who used the approach of Carrasco et al.^9^ to model ER reactions, but calculated the ER sticking coefficient from a formula dependent on the entropy and enthalpy of activation.

Engelmann et al.^15^ also developed a model to study plasma-catalytic NH_3_ synthesis, focusing on the surface kinetics. They included the same ER reactions as the aforementioned models, based on the model of Carrasco et al.^9^ However, they used a different approach for the calculation of ER reaction rate coefficients. Indeed, the ER rate coefficients were calculated in the same way as the LH rate coefficients, i.e., with the Eyring–Polanyi equation. Hence, the rate coefficients were dependent on the enthalpy and entropy of activation. For the ER reactions, the enthalpy of activation was assumed to be zero, while the entropy of the gas-phase species was assumed to be lost in the transition state (TS). Hence, the energy barriers and rate coefficients of the ER reactions were independent of the adsorbate and catalyst, i.e., the metal surface. They found that ER reactions of the H(g) + NH_x_* type were vital for NH_3_ formation on less noble catalysts, e.g., Fe, while on more noble catalysts, e.g., Ag, ER reactions did not significantly influence NH_3_ formation. ER reactions of the H* + NH_*x*_(g) type were found to be less important on all metals. Interestingly, they also noted that these reactions are less likely from a stereochemistry viewpoint. Furthermore, their model predicted that all metals yield similar NH_3_ production rates. In a later study by Gorbanev et al.,^16^ the model was validated with experiments, where it was also found that different metals show a similar activity. The same observations were also made in other experiments.^17−20^

Loenders et al.^21^ developed a kinetic model for plasma-catalytic partial oxidation of CH_4_ on Pt(111) and modeled the ER reactions in a manner similar to Engelmann et al.^15^ They included CH_4_(*g*) + O* → CH_3_* + OH* and CH_4_(*g*) + OH* → CH_3_* + H_2_O* in their reaction set. However, contrary to Engelmann et al., these reactions have enthalpy barriers and only the translational entropy was assumed lost in the transition state (TS), not the entire entropy. They found that, above 1000 K, the ER reactions were mainly responsible for CH_4_ dissociation. They also implemented some other ER reactions, namely, CH_3_(*g*) + O* → CH_3_O*, H(g) + O* → OH*, and O(g) + C* → CO*, to illustrate their potential. They reported that, if the enthalpy barriers of these reactions are set to 0 eV, the production rate of certain species, e.g., CH_3_OH, is enhanced.

Maitre et al.^22^ explored the plasma-catalytic nonoxidative coupling of methane with a kinetic model. They included ER reactions of the type C_*x*_H_*y*_(g) + C_*z*_H_q_* → C_*x*_H_*y*+1_(g) + C_*z*_H_q-1_* and the reverse reactions. They calculated the reaction rate coefficients for these reactions in a similar manner as Engelmann et al.^15^ Contrary to Engelmann et al.,^15^ however, the pre-exponential factor was calculated from collision theory and multiplied with a sticking coefficient that was set to 1 for all ER reactions. Their model predicted that the ER reactions of CH_3_(*g*) with CH_x_* species contributed to the formation of CH_4_.

Du et al.^23^ investigated plasma-catalytic CO_2_ hydrogenation over Ni and Cu. Their model included several ER reactions, e.g., H(g) + C* → CH*, H(g) + OH* → H_2_O*, and H(g) + CH_3_O* → CH_3_OH*. They modeled the ER reactions in the same way as Carrasco et al.^9^ and used the same coefficients for the calculations of the ER rate coefficients. It is noteworthy that these coefficients were used by Carrasco et al.^9^ to describe ER reactions in NH_3_ synthesis, while Du et al.^23^ used them for ER reactions in CH_4_ and CH_3_OH formation. They found that, on a Ni catalyst, CH_4_ is mainly produced by the ER reaction H(g) + CH_3_* → CH_4_(*g*), while on a Cu catalyst, H(g) + CH_3_O* → CH_3_OH* is mainly responsible for the production of CH_3_OH.

In summary, most of the studies discussed here attribute an important role to ER reactions in plasma catalysis. It is worth noting that some studies^10,15,21^ clearly mention that this is only true under certain conditions or assumptions, e.g., if the barriers for ER reactions are 0 eV. We need to make some remarks on how these ER reactions were modeled:

1. The parameters to determine the ER rate coefficients were nearly always estimated or derived from experimental fits.^9−12,22,23^ Fitting to experiments is difficult in plasma catalysis, as there are many possible underlying mechanisms that could have the same effect, e.g., the lack of a difference in metal activity found in NH_3_ synthesis could also be caused by the fact that all metals modulate the physical plasma characteristics in a similar way.^17^ This also explains why experimental validation of the kinetic models is difficult. The above estimations are of course necessary due to a lack of fundamental studies.
2. The product of the ER reaction is often assumed to be the one leading to the desired product, while possible byproducts are often neglected.^9−13,23^ For instance, H(g) + CH_3_* can only lead to CH_4_ formation.^23^
3. Because reaction rate coefficients for ER reactions often did not depend on the adsorbate involved^9−13,15,23^ or the ER sticking coefficient was the same for different adsorbates, ER reactions of a H radical with, e.g., N* and NH_2_* were equally likely to occur. Intuitively, one would expect the ER reaction with N* to be more likely, as N is not shielded by H atoms, in contrast to NH_2_*.

It is thus clear that, for a better understanding of ER reactions in plasma catalysis, more input is needed from fundamental studies in higher level models.

### 2.2 Fundamental Studies of Eley–Rideal Reactions

Some ER reactions have already been studied with fundamental methods, like MD, DFT, and quasi-classical trajectory calculations. The most studied ER reactions are H/H* and N/N* recombination to H_2_(*g*) and N_2_(*g*), respectively.^24−30^ Most interesting in the context of this paper is that, in general, hot atom formation is found to be more important than ER reactions, and the importance of ER reactions increases with increasing coverage.^24−28^ Also, energy loss through electron–hole pair excitation was found to be important, especially for H atoms.^29,30^ Lastly, stereodynamics were also found to play a significant role, especially for N.^29^

Zhou et al.^31^ investigated the reaction mechanism of the ER reaction D(g) + CD_3_* at Cu(111) with *ab initio* MD (AIMD). They reported that, under the conditions investigated, 3% of their trajectories lead to CD_4_ formation via ER, 4.7% to D_2_ formation via ER, 3.4% lead to CD_4_ formation via an HA mechanism, and 88.9% lead to adsorption or reflection. Zhou et al.^32^ also performed AIMD simulations for H(g) + Cl* at Au(111). They found that the production of HCl through the HA mechanism was more likely than adsorption of the H radical and that electron–hole pair excitation only had a minor effect.

Lin et al.^33^ investigated the reaction H(g) + CO_2_* on a Ni(110) surface with MD. For both low- and high-energy H atoms, the HA mechanism was dominant over the ER mechanism. At higher coverages, the importance of ER reactions increased. Both ER and HA reactions resulted in different products. Lin and Schatz^34^ also investigated the reaction CH_2_(*g*) + CO_2_* on a Ni(110) surface with MD and found that roughly 45% of their trajectories resulted in a reaction between CH_2_ and CO_2_ leading to different products. These reactions were found to proceed mostly through the ER mechanism.

Similarly, Zhou et al.^35^ studied the reaction H(g) + CO* on a Cu(111) surface with AIMD. They found that, for low-energy H atoms, over half of their trajectories lead to reflection, while for highly energetic H atoms, adsorption was most common. No ER reactions were found. Interestingly, they also reported that, in ca. 5% of the trajectories, the H impinging on the surface leads to displacement of the CO* molecule, i.e., CO stays adsorbed but moves to another adsorption site. This process was found to proceed via an HA mechanism. Wu et al.^36^ investigated the reaction O(g) + CO* on a Pt(111) surface with AIMD simulations. Most of their trajectories lead to CO_2_ formation via an HA mechanism. They explained this through stereodynamics, i.e., the C atom is shielded by the O atom from above, which causes the C atom to be only accessible from the surface.

It is worth noting that all these studies are not performed in the context of plasma catalysis, and thus, the conditions used in the simulations do not necessarily mimic those of plasma catalysis.

There are very few fundamental studies of ER reactions in the context of plasma catalysis. Yamijala et al.^37^ investigated ER reactions involving N, H, and NH on Pt(111) and Cu(111) with AIMD. They first studied the stability of N- and H-terminated surfaces at 300 K, as they assumed that these surfaces are a good representation of the plasma catalysis environment. They found that, on Cu(111), only the H-terminated surface was stable, while for Pt(111), both cases were stable. For Cu(111), they simulated what happened when a N atom impinges on the H-terminated surface and found that this mostly leads to the formation of gaseous NH_2_ and NH_3_. On Pt(111), both the impingement of an H and N atom on the N- and H-terminated surfaces did not lead to any product formation.

Yi et al.^38^ studied ER reactions of the type, NH_*x*_(g) + CH* → H_*x*_NCH* and NH_x_* + CH(g) → H_*x*_NCH*, with nudged elastic band (NEB) calculations, to find the minimum energy path. They found that all ER reactions were nonactivated. Cui et al.^39^ studied ER reactions between an H radical and several adsorbates, e.g., CO_2_*, HCOO*, and CH_3_O*, in the context of CO_2_ hydrogenation over a Cu cluster supported on Al_2_O_3_. They used the same method as Yi et al.^38^ and found that most ER reactions studied had no barrier or a reduced barrier compared to the LH reactions. Hence, they concluded that ER reactions can help facilitate plasma-catalytic CO_2_ hydrogenation. It is important to note that adsorbates were located at the Cu–Al_2_O_3_ interface in their calculations, and thus, their geometries were different from adsorbates on a planar metal surface. Furthermore, NEB calculations do not include dynamical effects, which are typically considered to be important for ER reactions,^7^ i.e., NEB calculations show that ER reactions can happen through a reaction path without a barrier, but do not tell us anything about the probability that this reaction actually happens, e.g., adsorption might be more likely than the reaction.

Although the discussion above covers different systems, some general conclusions regarding ER and HA reactions between a gaseous species and adsorbate can be made. First, as expected, the surface coverage is an important factor in determining the importance of ER reactions. Second, in most cases, adsorption or reflection is preferred over ER and HA reactions, sometimes even when the surface is completely covered with the adsorbate. For instance, even for an Ag(111) surface completely covered with N atoms, only 35% of incident N will recombine to N_2_.^25^ This is not incorporated in most kinetic models, where it is often implicitly assumed that each collision will lead to an ER reaction. Furthermore, the reaction between the adsorbate and gaseous species can lead to different products, as illustrated by the reactions of CH_2_(*g*) + CO_2_*^32^ and D(g) + CD_3_.^31^ The impingement of the gaseous species can also lead to desorption of the adsorbate, which can be regarded as another product. This distribution of products is not included in most kinetic models. Lastly and perhaps most importantly, stereodynamics are crucial in ER reactions.^7^ Stereodynamics can also lead to a preference for the HA mechanism, as illustrated by the reaction of CO* + O(g).^32^

## 3 Aim of This Work

In summary, kinetic models have been used to model ER reactions in plasma catalysis. The ER reactions are either modeled through a barrier or some kind of sticking coefficient, or sometimes a combination of both. These kinetic models conclude that ER reactions are beneficial for the considered process and sometimes even suggest that the ER mechanism is the sole mechanism responsible for the formation of the desired product.^23^ Several ER reactions have already been investigated with more fundamental methods like MD. From these fundamental studies, it is clear that several assumptions in the kinetic models contradict the MD results.

Consequently, our understanding of ER reactions and especially how to include them in kinetic models in the context of plasma catalysis needs to be improved. Hence, in this work, we will construct intersections of a potential energy surface (PES) for a series of ER reactions from DFT calculations. These PES intersections will be constructed as a function of the parallel distance between a H atom and adsorbate and the height relative to the adsorbate, similar to elbow plots for chemisorption reactions. We opt for this approach because it is computationally relatively cheap and thus allows for studying multiple reactions. We will limit ourselves to studying ER reactions where the gas-phase species is a H atom, for the sake of simplicity. We study these reactions on Ni(111), Cu(111), and Ru(0001) surfaces, as these are commonly used catalyst materials. To study the effect of the metal on the PES intersection, we also include Ti(0001) and Au(111) as representatives for a very strongly and very weakly binding metal, respectively. This range of metals allows us to obtain a clear picture whether and how the metal affects the PES profile. Also, we will investigate how the PES is influenced by the coverage. To the best of our knowledge, it is the first time that a range of ER reactions are systematically studied in the context of plasma catalysis. Furthermore, it is also the first time that the PES approach is applied in this context.

Based on the discussion of the PES results and the fundamental studies reported above, we will make recommendations for the kinetic modeling of these reactions. We emphasize that the goal of this paper is qualitative in nature, i.e., the goal is not to calculate a rate coefficient for each ER reaction studied, because obtaining a numerical value would require AIMD simulations for each reaction on every metal surface, which is too computationally expensive. Rather, we aim to make some general observations on ER reactions relevant in plasma catalysis and to provide recommendations for implementing ER reactions in kinetic models. These recommendations deviate from what is currently used in kinetic models for plasma catalysis.

## 4 Method

### 4.1 DFT Setup

Periodic plane-wave DFT calculations were carried out using the Vienna *Ab initio* simulation Package (VASP, version 6.2.1).^40−45^ The Bayesian error estimation functional with van der Waals correction^46,47^ (BEEF-vdW) was used as density functional. The core electrons were described by the projector augmented wave method.^48,49^ A plane-wave kinetic energy cutoff of 400 eV was used for the plane-wave basis set, and the energy in the self-consistent field was converged to within 10^–5^ eV. Spin polarization was taken into account for all calculations involving the Ni surface or a gaseous H atom, i.e., for construction of the PES.

The lattice constants were optimized using a Γ-centered 20 × 20 × 20 k-point mesh. The force on each atom was converged within 0.005 eV/Å. The lattice constants for Cu, Ni, and Au were 3.66, 3.53, and 4.20 Å, respectively. This is in good agreement with the experimental values of 3.60, 3.50, and 4.07 Å, respectively.^50^ Ru and Ti have an hcp lattice structure, and thus, two lattice constants characterize their structure. For Ru, they were found to be 2.72 and 4.29 Å; for Ti, the calculated values were 2.92 and 4.62 Å. This is in agreement with the experimental values of 2.70 and 4.28 Å for Ru and 2.95 and 4.69 for Ti.^51^

All metal surfaces, i.e., the Ni, Cu, and Au FCC(111) surfaces and the Ru and Ti HCP(0001) surfaces, were modeled as 3 × 3 periodic 4-layer slabs with a 15 Å vacuum region separating the periodically repeated slabs. During geometry optimizations, the two upper layers and adsorbates were fully relaxed, while the lower layers remained fixed at the equilibrium bulk positions. A Γ-centered 4 × 4 × 1 k-point mesh was used for sampling the Brillouin zone. The force on each atom was converged to within 0.005 eV/Å. The interlayer distance was optimized with these settings, where the only difference from optimizations that include an adsorbate on the surface is that only the *Z* coordinate is allowed to relax. The interlayer distance between the two top layers decreased with 0.63, 0.72, 3.18, and 6.72% for Cu, Ni, Ru, and Ti, respectively. The interlayer distance between the top two layers increased with 2.45% for Au. Convergence testing of computational parameters can be found in Section S.1 of the Supporting Information.

### 4.2 Construction of PES Intersections

**Figure 2:**
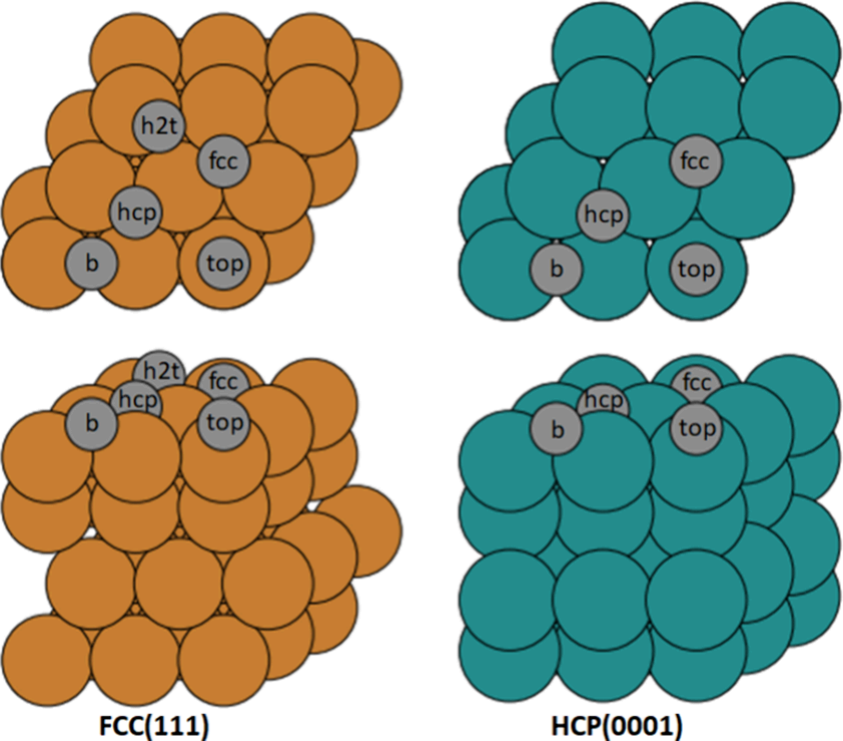
Top (top panel) and side (bottom panel) views of FCC(111) slab, cfr. Cu, Ni, and Au (left side). Top and side views of HCP(0001) slab, cfr. Ru and Ti (right side). High-symmetry sites are indicated by gray circles, b is short for bridge site, and h2t is short for hcp-to-top site.

In Table 1, we present an overview of the ER reactions and surfaces that are investigated. The reactions and metal surfaces were chosen based on reactions included in the kinetic models discussed in Section 2.1 above. Some reactions are investigated on multiple surfaces to evaluate how the metal influences the PES. The last column indicates if and on which metal surface this ER reaction was investigated at a higher coverage of the adsorbate. This high coverage was realized by placing an adsorbed molecule on all equivalent adsorption sites on the surface. As we are working with a 3 × 3 slab, this means that, for high coverage, all 9 equivalent high-symmetry sites are occupied by an adsorbate. For instance, for O, all 9 fcc sites on the Ni(111) surface were covered with O atoms. Prior to the PES intersection calculations, each adsorbate is optimized on the metal surface. The most stable adsorption site for each adsorbate and metal can be found in Table 1 between brackets. Figure 2 depicts all high-symmetry sites on the FCC(111) and HCP(0001) surfaces.

**Table 1:** Overview of the Studied Systems

	metal surface					
ER reaction	Ni(111)	Cu(111)	Au(111)	Ru(0001)	Ti(0001)	investigated at high coverage?
O* + H(g)	√ (fcc)					Ni(111)
OH* + H(g)	√ (fcc)					
C* + H(g)	√(hcp)	√ (fcc)	√ (fcc)	√ (hcp)	√ (fcc)	
CH* + H(g)	√ (fcc)					Ni(111)
CH_2_* + H(g)	√ (fcc)					
CH_3_* + H(g)	√ (fcc)	√ (hcp)	√ (top)	√ (fcc)	√ (fcc)	
N* + H(g)				√ (hcp)		Ru(0001)
NH* + H(g)	√ (hcp)	√ (hcp)	√ (fcc)	√ (fcc)	√ (fcc)	Ru(0001)
NH_2_* + H(g)				√ (bridge)		Ru(0001)
CO* + H(g)	√ (fcc)					
CH_3_O* + H(g)		√ (fcc)				

The PES intersections, or elbow plots, are constructed by calculating the energy of the system for different positions of the H atom relative to the adsorbate, as illustrated in Section S.2 of the Supporting Information. The different positions of the H atom are chosen as follows: (1) A set of points is chosen along a line connecting the adsorption site of the adsorbate with other high-symmetry sites on the metal surface. The points are chosen so that the first point is at the adsorbate, the last point is at the end of the line, and the spacing between these points is ca. 0.2 Å. These points determine the *X* and *Y* coordinates of the H atoms. (2) At each of these points, a set of heights, or *Z* coordinates, is chosen so that the highest *Z* coordinate is at least 2 Å above the top of the adsorbate. The spacing in this direction is 0.25 Å. (3) For each combination of *X*,*Y* coordinates with a *Z* coordinate, the total energy of the system is calculated when the H atom is located at this position. In this manner, we obtain a set of energies corresponding to different positions of the H atom relative to the adsorbate. All these positions are located in a plane perpendicular to the surface. An example of this is depicted in Figure S.5. Each position of the H atom for which the total energy is calculated is represented by a white sphere in Figure S.5. We chose to only calculate the PES along certain lines on the surface, as we are studying flat surfaces and the PES is mostly symmetric.

The energy is always plotted relative to the energy of the system when the H atom is located far away from the adsorbate and surface, i.e., the elbow plots show the relative stability of a certain position of the H atom. This reference energy is calculated as the total energy of the system when the H atom is located midway between periodic slab images in the *Z* direction.

## 5 Results and Discussion

We first briefly discuss the general features of the PES intersections. Some figures contain multiple panels, which depict PES intersections along different lines on the surface for the same system. The height, i.e., the *Z* coordinate and *XY* distance of the H atom are relative to the position of the atom through which the adsorbed is bound to the surface. Areas where the energy is comparatively high are white, to ensure proper color scaling. In areas delimited by a dashed line, the relative energy is negative, i.e., when the H atom is located in this area, the system is stable relative to the system in which the H atom is far away from the surface. In areas delimited by a full line, the system is unstable relative to the reference system. The reader should be aware that between different figures a different color scaling can be used, but for different panels in the same figure, the scaling is always the same. It is also worth noting that these figures are the result of static DFT calculations. In reality, dynamical effects can change results, but taking this into account would require AIMD simulations, which is intractable to investigate for all ER reactions studied here.

### 5.1 Monoatomic Adsorbates

**Figure 3:**
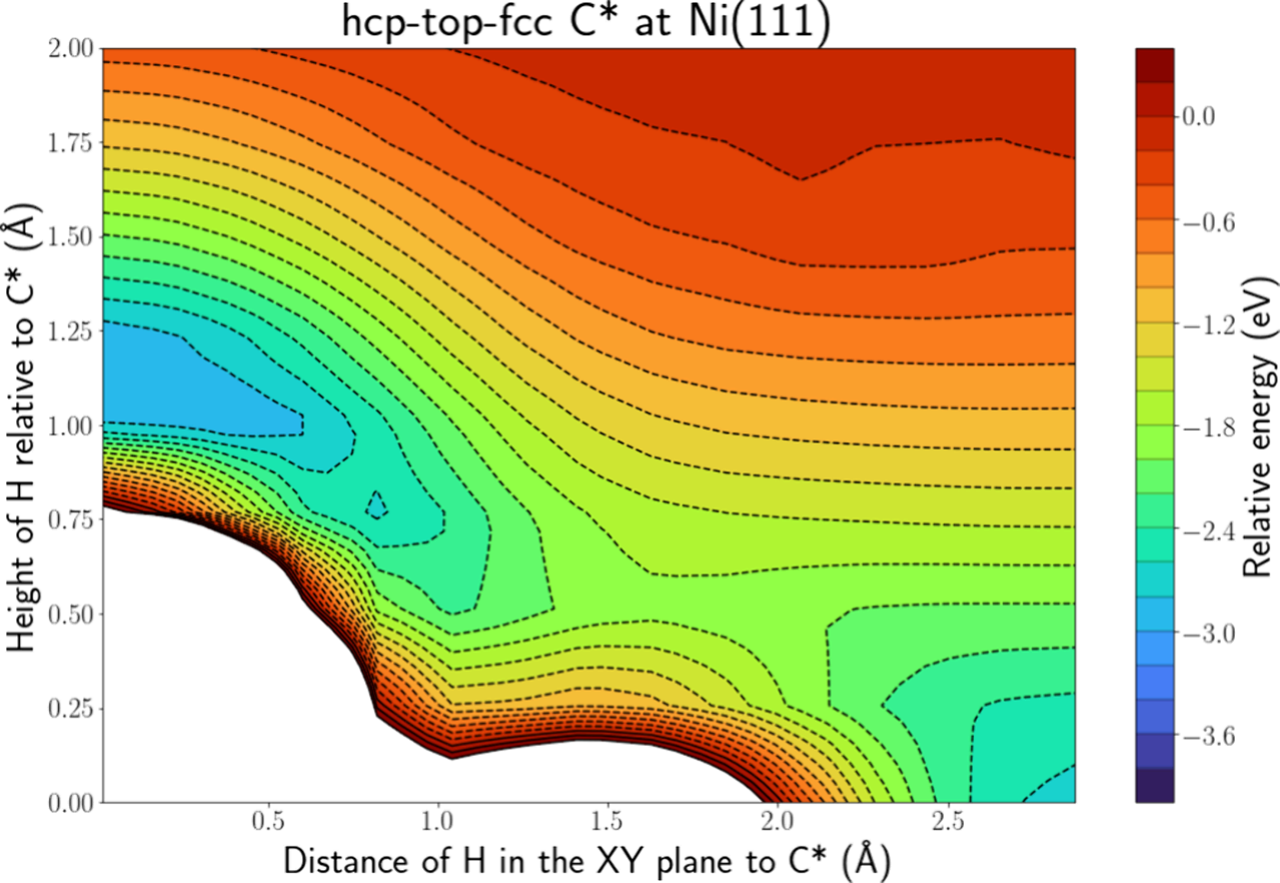
PES intersection for H(g) + C* at the Ni(111) surface along the hcp-top-fcc line.

Figure 3 depicts the PES intersection for H(g) + C* at Ni(111) along the hcp-top-fcc line, while Figure S.6 depicts the PES along the hcp-bridge-fcc line, which shows a similar profile. Both figures show a similar picture. We can clearly see that there are two energy wells for the H atom. One is located around the top side of the C atom at a distance of ca. 1.1 Å. This distance corresponds to the C–H bond length in CH* found in our calculations and thus corresponds to CH* formation. The second area is located close to the surface and at *XY* distances greater than 2.0 Å. Thus, it corresponds to H adsorption at the fcc site. Both wells are accessible without a barrier. We can conclude that ER-assisted formation of CH is certainly possible and barrier-free for this system. It is clear that the *XY* coordinate of the H atom, once it is close enough to the surface, will decide whether an ER reaction happens. Namely, we can imagine a vertical line at an *XY* coordinate of ca. 1.6 Å dividing the hcp-top-fcc PES in two: if the *XY* distance between the H and C atom is shorter than 1.6 Å, an ER reaction can occur, while if the *XY* distance is greater, the H atom can adsorb. Both the adsorption and ER well are relatively deep, i.e., ca. 3 eV. The adsorption well will be even deeper closer to the surface. While 3 eV is only a rough estimate, we can safely state that both the ER reaction and adsorption will be strongly exothermic. For adsorption, this energy can easily be lost through electron–hole pair excitation and energy exchange to phonons, although the latter might be slow due to the large mass mismatch. For the ER reaction, it is more difficult to dissipate this energy and it is quite possible that this energy release will lead to the breaking of the C–H bond. It is also clear that the H atom can diffuse from the adsorption well to the energy well associated with CH* as both wells are connected. However, this will be associated with a barrier of ca. 0.8 eV. This barrier might be overcome by virtue of the energy that is released when the H atom approaches the surface. This process corresponds to an HA mechanism.

The PES intersections for H(g) + N* at Ru(0001) and H(g) + O* at Ni(111) are shown in Figures S.7 and S.8, respectively. Both look similar to the PES intersection for H(g) + C* at Ni(111). The depth of the wells associated with NH* and OH* formation is 2.4 eV. The imaginary line separating the ER well from the adsorption well along the hcp-top-fcc line is located at similar *XY* distances, ca. 1.3 Å for H(g) + N* and ca. 1.4 Å for H(g) + O*.

In summary, we conclude that the ER reactions with atomic adsorbates studied here have no enthalpy barrier, as is often assumed in plasma-catalytic kinetic models.^15,21^ Furthermore, the difference between the various atomic adsorbates (C*, N* or O*) is minimal, and thus, using similar numerical values for the rate coefficients of ER reactions with different atomic adsorbates seems reasonable. However, we cannot claim for sure that ER reactions can actually happen, as the ER reaction is strongly exothermic. This exothermic energy needs to be dissipated quickly enough or else the formed bond might be broken again. The adsorbate might also desorb before significant energy dissipation or chemical reaction has taken place. However, the desorption rate will depend on the stability of the adsorbate in the gas phase and is likely to only affect certain adsorbates, (e.g., H_2_, NH_3_, and CH_4_). One possible energy dissipation channel is phonon excitation, but this channel is likely to be comparatively slow due to the large exothermicity compared to the energies involved in phonon excitations. For similar reasons, we also expect dissipation through electron–hole pair excitation to be comparatively slow. The remaining channels are rotational and vibrational excitation of the adsorbate. Again, we do not expect rotational excitation to affect the results considerably. On the other hand, vibrational excitation should affect results considerably, because vibrationally excited bonds also dissociate more readily. Since lifetimes of vibrationally excited adsorbates are often significant, the probability of an ER reaction followed by vibrational excitation and subsequently by dissociation might be significant.

### 5.2 Polyatomic Adsorbates

**Figure 4:**
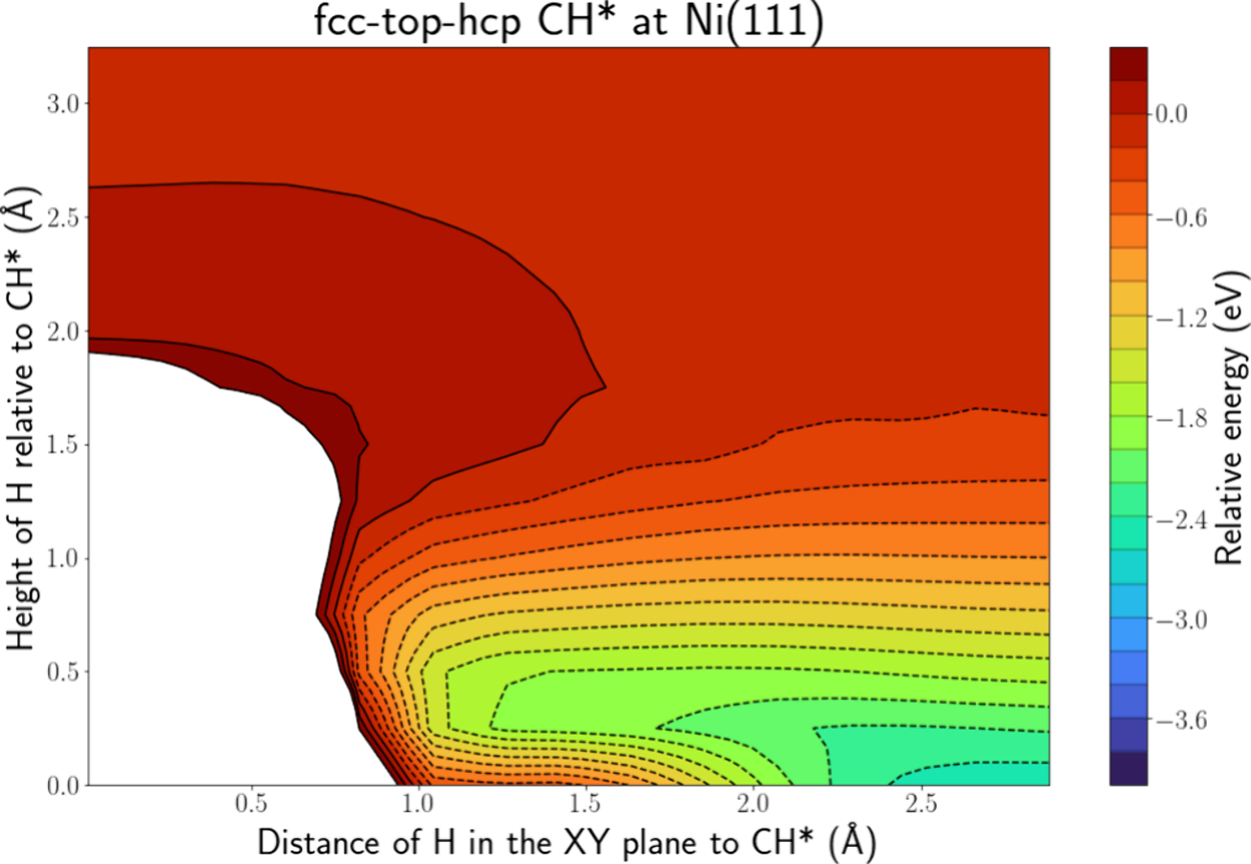
PES intersection for H(g) + CH* at the Ni(111) surface along the fcc-top-hcp line.

Figure 4 depicts the PES intersection for H(g) + CH* at Ni(111) along the fcc-top-hcp line, while Figure S.9 shows the PES along the fcc-bridge-hcp line, which shows a similar profile. In contrast to the PES intersections for the atomic adsorbates, now only the energy well associated with adsorption is present. On the top side of CH*, the H atom is repelled, and there is no area associated with CH_2_* formation. Hence, it is clear that this ER reaction will be very difficult, as the most stable CH conformation does not allow for ER reactions, due to the H atom on top of the C atom causing sterical hindrance. The adsorption well extends toward the C atom to distances of ca. 1.2 Å from the C atom, indicating the formation of CH_2_* through an HA mechanism. Thus, based on the PES intersection, we find adsorption and the formation CH_2_* through an HA mechanism possible and far more likely than an ER mechanism. While the latter seems improbable based on the PES intersection, we do not completely rule out the possibility, as a different conformation of CH* will affect the results.

**Figure 5:**
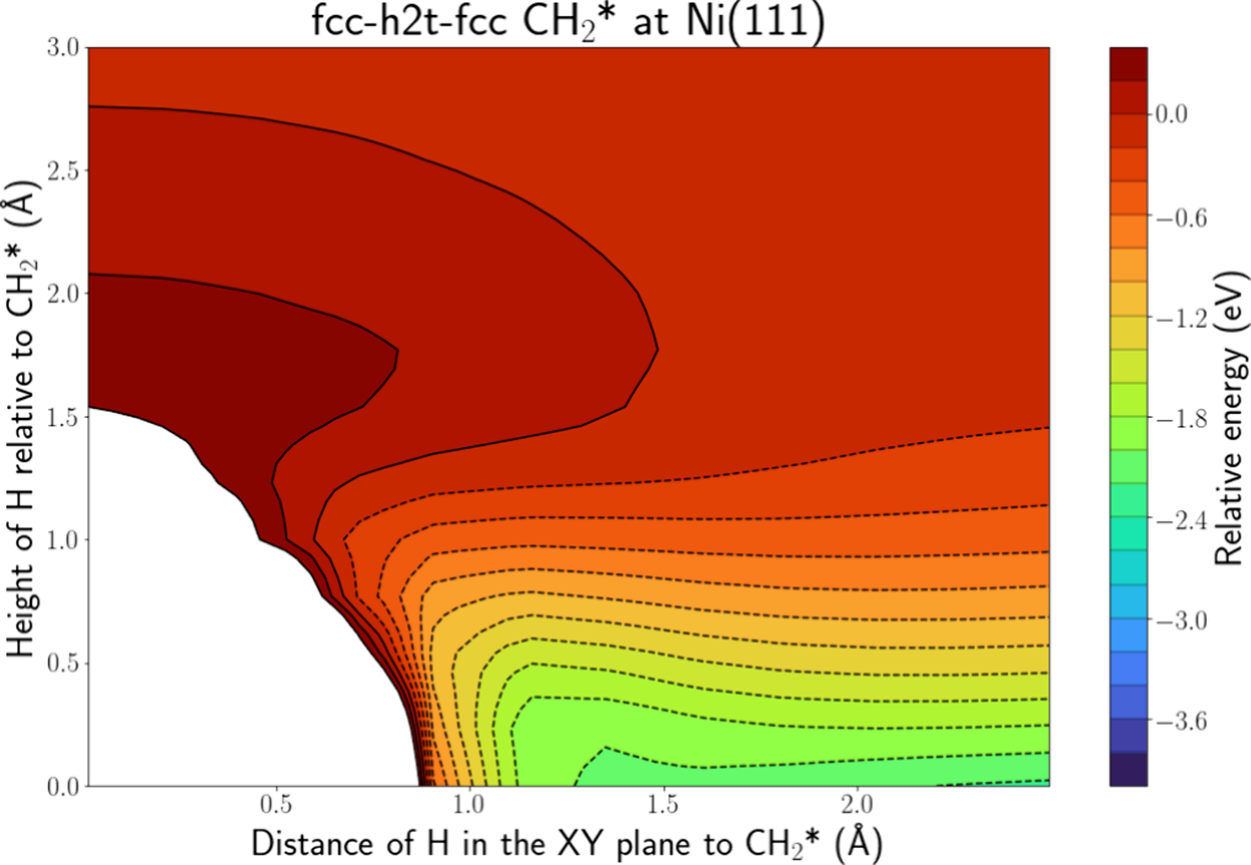
PES intersection for H(g) + CH_2_* at the Ni(111) surface along the fcc-h2t-fcc line.

Figure 5 depicts the PES intersection for H(g) + CH_2_* at Ni(111) along the fcc-h2t-fcc line. The corresponding PES intersections along the fcc-top-hcp and fcc-bridge-hcp lines are depicted in Figure S.10. One of the H atoms of CH_2_* is located along the latter two lines, while the fcc-h2t-fcc line is located right in between these lines. The interpretation is similar to CH*, i.e., adsorption and an HA mechanism are far more likely than ER. Somewhat surprisingly, the CH_2_* PES intersections along the fcc-bridge-hcp and fcc-h2t-fcc line show similar profiles, so the H atoms bound to the C atom also seem to have an influence on the profile along the fcc-h2t-fcc line, although there is no H atom along this line. This indicates that the sterical hindrance has further increased compared to CH* and that it will also be less likely to find CH_2_* in a conformation that will minimize sterical hindrance. Furthermore, on the intersection along the fcc-top-hcp line, the adsorption well does not reach toward the C atom of CH_2_*. This is due to one of the H atoms of CH_2_* being located directly above the Ni atom at the top site. There is no room in between, these two atoms to reach the C atom in this direction. This again illustrates that stereodynamics are important.

The PES intersections for H(g) + NH* at Ru(0001), H(g) + OH* at Ni(111), H(g) + NH_2_* at Ru(0001), and H(g) + CH_3_O* on Cu(111) are shown in Figures S.11–S.14, respectively. The discussion of these figures is similar to the discussion in the two paragraphs above: adsorption is more likely than HA, which in turn is more likely than ER, and stereodynamics are important.

**Figure 6:**
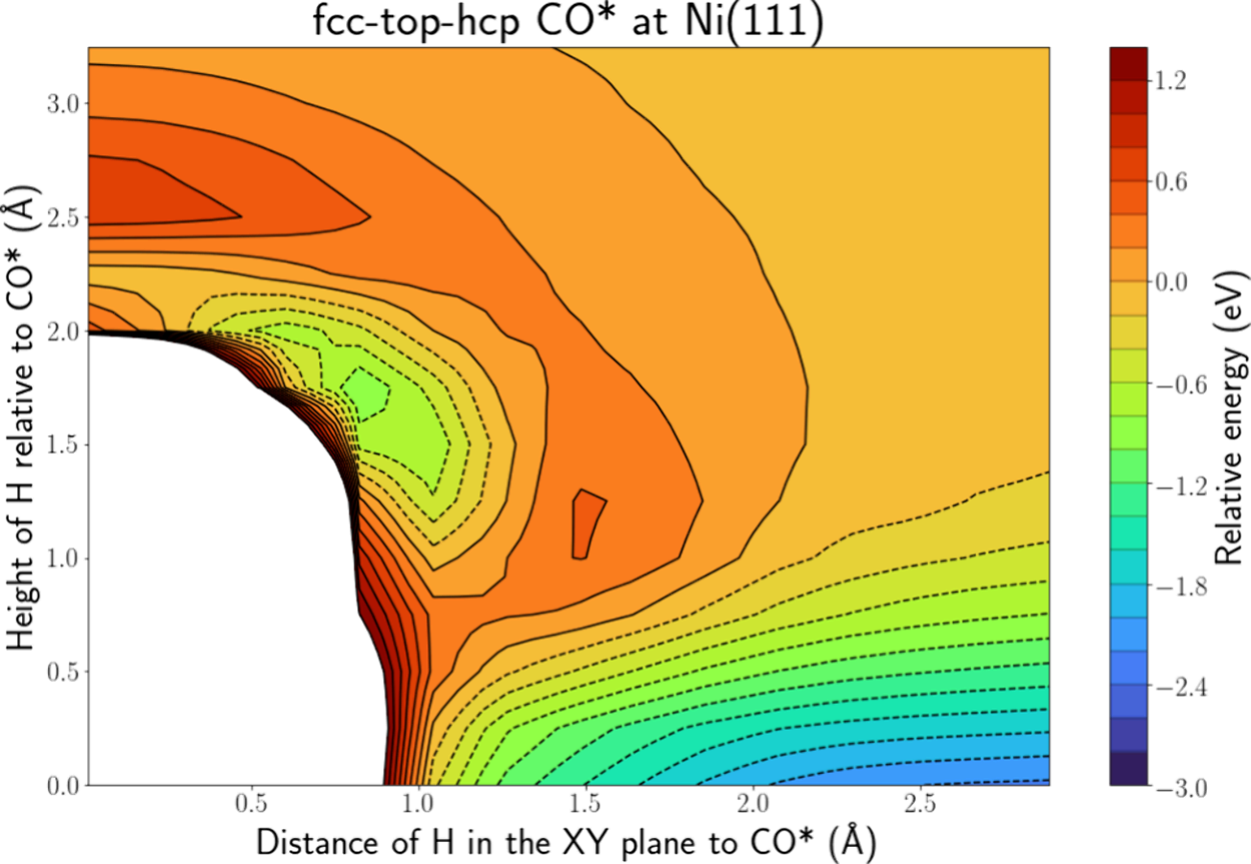
PES intersection for H(g) + CO* at the Ni(111) surface along the fcc-top-hcp line.

Figure 6 depicts the PES intersection for H(g) + CO* at Ni(111) along the fcc-top-hcp line, while Figure S.15 shows the PES along the fcc-bridge-hcp line, which shows a similar profile. There is again an energy well associated with adsorption close to the surface that extends toward the C atom of CO*. The furthest edge of the adsorption well is ca. 1.1 Å away from the C atom. This could be an indication for an HA mechanism leading to HCO* formation. In contrast with the other polyatomic systems, this PES also shows a second energy well, located ca. 1 Å from the O atom of CO. Hence, we associate this well with COH* formation. However, this energy well is surrounded by a barrier. Given that the adsorption energy well is not surrounded by a barrier, we consider adsorption more likely than an ER reaction.

When we compare our findings for H(g) + CO* at Ni(111) to the findings of Zhou et al.^35^ who studied H(g) + CO* at Cu(111) with AIMD, there are similarities. As discussed in Section 2.2, none of their trajectories led to an ER reaction, while adsorption, as well as reflection, was found to be far more likely. Furthermore, in 5% of their trajectories, the CO molecule was displaced. In some cases, this was found to proceed through an HCO* intermediate formed via an HA mechanism. These similarities are encouraging, as it shows that the PES intersections can give us a good approximation for computationally more expensive AIMD simulations.

**Figure 7:**
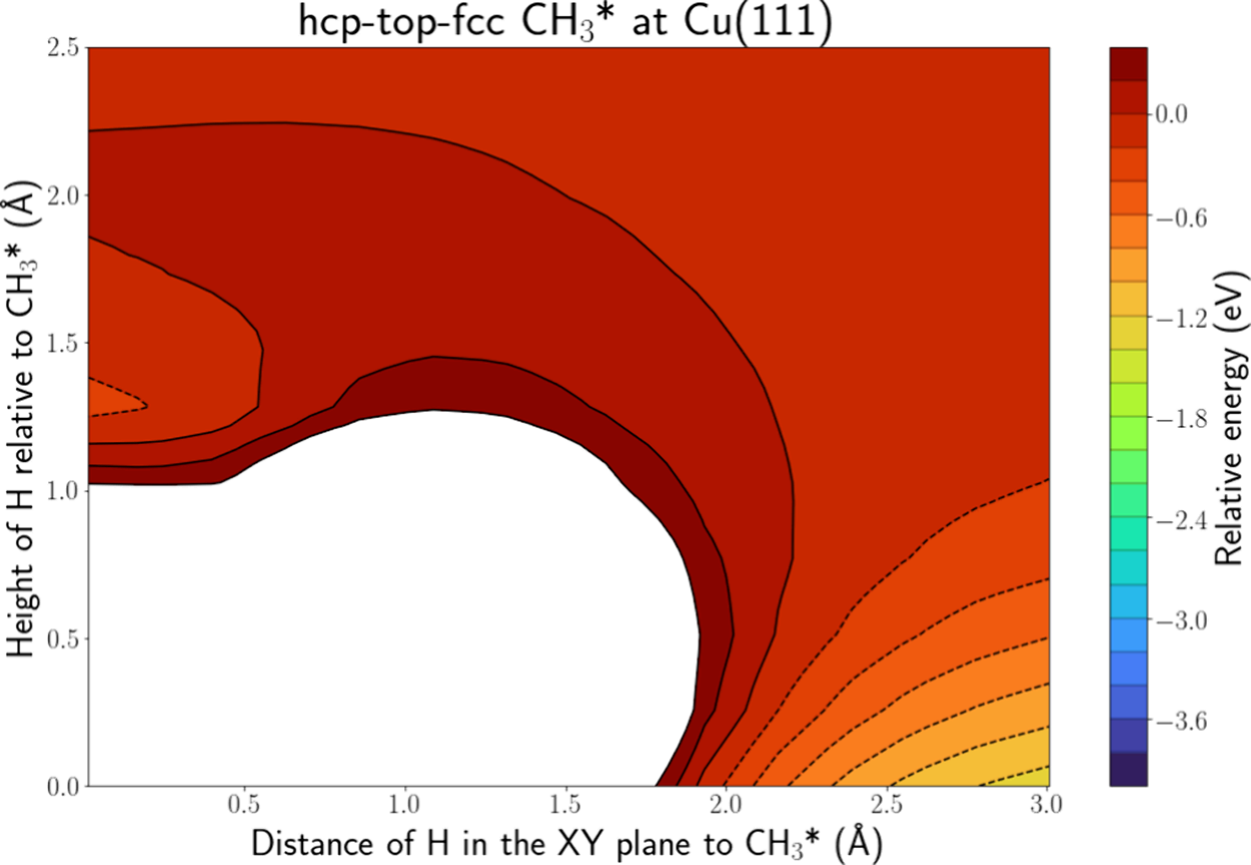
PES intersection for H(g) + CH_3_* at the Cu(111) surface along the hcp-top-fcc line.

Figure 7 depicts the PES intersection for H(g) + CH_3_* at Cu(111) along the hcp-top-fcc line, while Figure S.16 shows the PES along the hcp-bridge-fcc line. A H atom bound to C is present along the former line, while the latter line is located right in between two H atoms. Similar to CO*, there is an energy well present associated with an ER reaction, which could lead to both CH_4_ and H_2_ formation, as the distances to C and one of the H atoms are 1.25 and 1.32 Å, respectively. However, this area is again surrounded by a barrier. Furthermore, for both products to be formed, a bond needs to be broken, i.e., the C–H bond for H_2_ formation and the C-surface bond for CH_4_ formation. The energy well has a depth of only 0.2 eV, which is considerably less than the ER wells discussed above. Thus, there is only a small amount of energy released that could be used to break the C–H or C-surface bond. Hence, we consider adsorption to be much more probable.

Zhou et al.^31^ performed AIMD simulations for D(g) + CD_3_* at Cu(111). Almost 89% of their trajectories led to reflection or adsorption, while only 7.7% led to D_2_ or CD_4_ formation. This is in agreement with our results that predict adsorption to be far more likely than an ER reaction. Zhou et al.^31^ also reported that 3.4% of their trajectories led to CD_4_ formation via an HA mechanism. This cannot be derived from the presented PES intersections but might become visible when the PES intersections are expanded to the area between the C atom and surface.

In summary, while we find that ER reactions are possible for some of the polyatomic adsorbates studied here, we conclude that, in general, adsorption and/or an HA reaction are more probable than an ER reaction due to sterical hindrance.

### 5.3 Influence of the Metal

**Figure 8:**
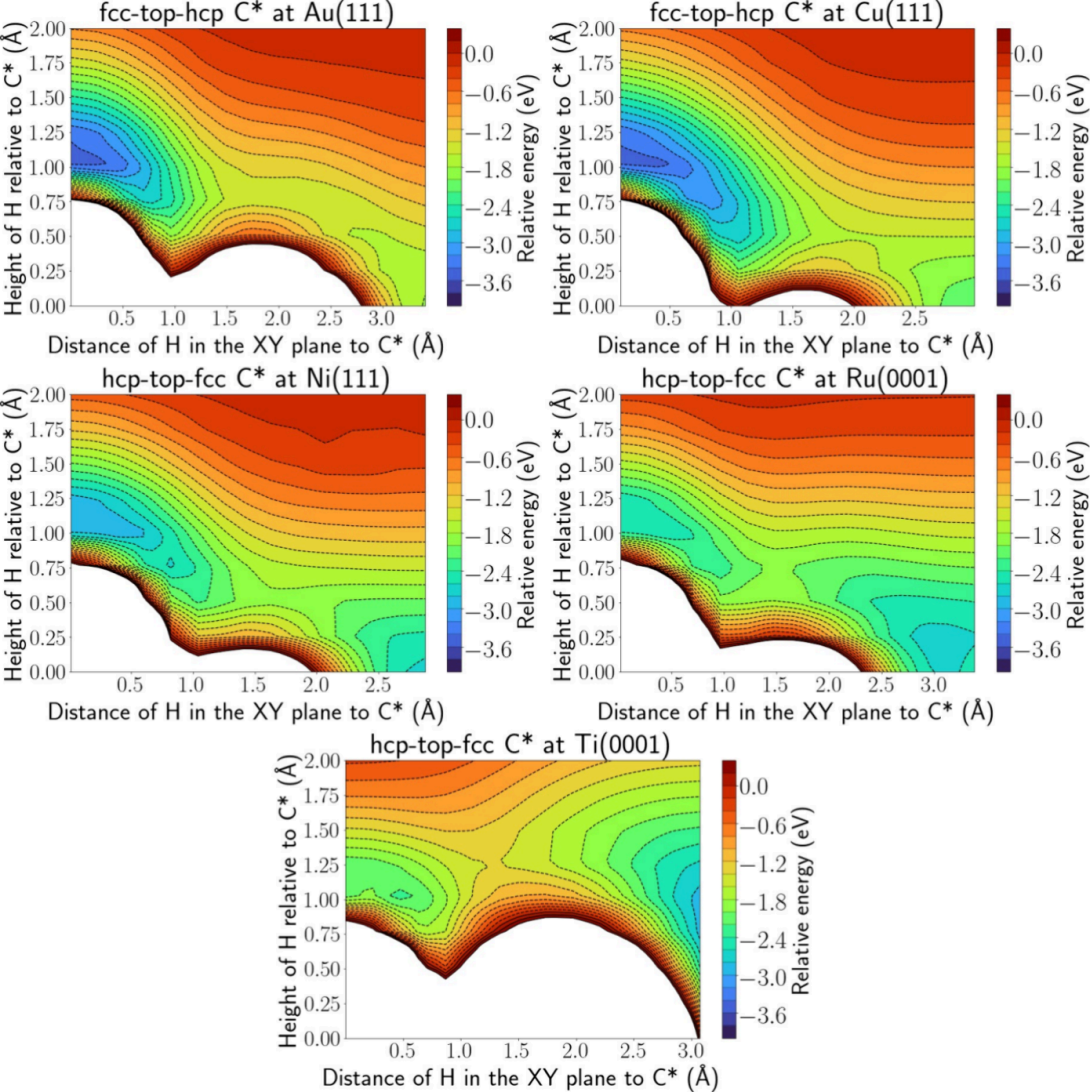
PES intersection for H(g) + C* at Au(111) (top left), Cu(111) (top right), Ni(111) (middle left), Ru(0001) (middle right), and Ti(0001) (bottom panel).

Figure 8 illustrates the PES intersections for H(g) + C* on all-studied metal surfaces. The picture looks similar for all five metals, but there are clear trends connected to the binding strength of the catalyst. In this case, the binding strength of each metal can be measured by the adsorption energy of the C atom. The stronger the bond between the adsorbate and catalyst surface is, the more negative the adsorption energy is. The C adsorption energies can be found in Section S.3 of the Supporting Information, and the absolute value has, as expected, the following trend: Au < Cu < Ni < Ru < Ti. It is clear from Figure 8 that, if the catalyst binds more weakly, the energy well associated with an ER reaction is deeper and wider (Au > Cu > Ni > Ru > Ti).

A similar observation is made for H(g) + CH_3_*, depicted in Figure S.17. The ER well has even completely disappeared for CH_3_* at the Ti(0001) surface. These trends have the opposite effect on the likelihood for ER reactions, i.e., the wider the well, the greater the chance that the H atom drifts into it, while the more energy is released upon reaction, the more likely the formed bond will immediately be broken to dissipate this energy. Nevertheless, if the metals have sufficiently different binding strengths, there will probably be an effect on the ER probability. For H(g) + NH*, shown in figure S.18, the adsorption energy well stretches out more toward NH* for the weaker catalysts, indicating that an HA reaction becomes more likely, although the trend is less pronounced than for the ER well in the case of C* and CH_3_*.

To definitively gauge the effect of the metal surface on the ER or HA reaction probability, AIMD simulations would be needed. However, our results suggest that the metal will influence the ER reaction probability, and that the assumption that ER reactions cause all metals to have the same activity, e.g., made by Engelmann et al.,^53^ might break down, despite the good agreement with experimental observations.

### 5.4 Influence of the Coverage

Figures S.19 and S.20 depict the PES intersections for H(g) + N* at Ru(0001) and H(g) + O* at Ni(111) for high coverage. This means that all 9 hollow sites in the supercell are occupied by an adsorbate. When we compare these profiles to their respective low coverage equivalents, Figures S.7 and S.8, i.e., when only 1 of the 9 equivalent high-symmetry sites is occupied, there are clear differences. The ER well becomes deeper, meaning that the ER reaction is more exothermic. Also, the adsorption well moves further away from the surface and is located right in between two adjacent atoms. Hence, it becomes more difficult for the H atom to reach the surface, as it might get trapped in the energy well between two adjacent adsorbates and subsequently move closer toward one of the adjacent atoms and bind to it. A barrier is associated with the latter step. Thus, we can conclude that, upon increasing coverage, the adsorption probability drops and the ER probability increases, although the ER reaction is still strongly exothermic so the dissipation of that energy might still lead to breaking of the bond. Hence, the ER probability is likely to remain smaller than 1.

**Figure 9:**
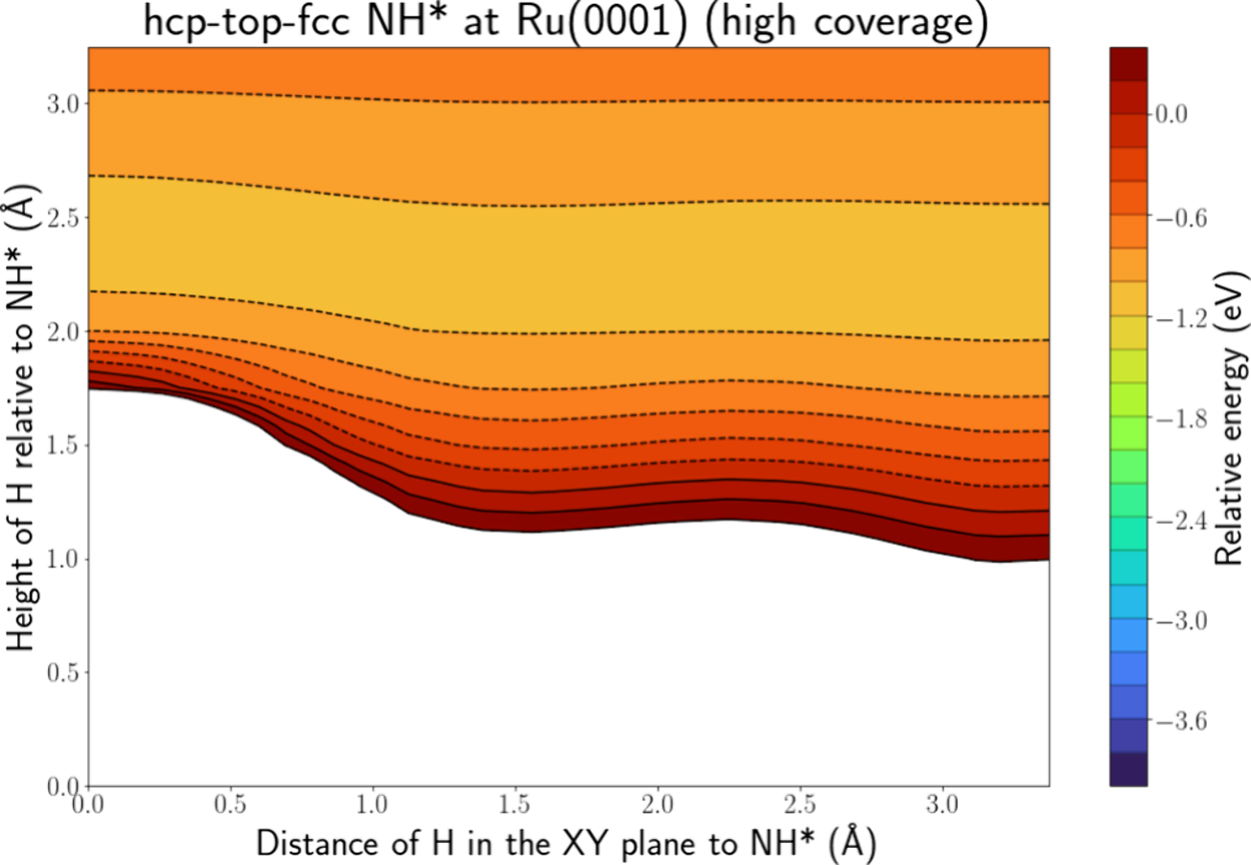
PES intersection for H(g) + NH* at the Ru(0001) surface along the hcp-top-fcc line for a high coverage of NH*.

More interesting are Figure 9 and Figure S.21 depicting the PES intersections along the hcp-top-fcc and hcp-bridge-fcc lines for H(g) + NH* at Ru(0001) when all 9 hcp sites are occupied by NH*. This PES intersection is drastically different compared to a single adsorbed NH* molecule, depicted in Figure S.11. There is now only one energy well, i.e., a broad band located ca. 2.3 Å above the adsorbate. This energy well is too far from the N atom, i.e., 2.3 Å, to be associated with NH_2_* formation. It is probably associated with N* and H_2_ formation, as it is only located ca. 1.3 Å from the H atom of NH*. It has to be noted that the breaking of the N–H bond will have a barrier, not captured on the PES intersection, but it is possible that the energy released upon entrance of the H atom into the well can be used to surmount this barrier. Similar to the atomic adsorbates, the surface is now more difficult to reach and thus adsorption, and also HA formation, is less likely. The PES intersections for H(g) + NH_2_* at Ru(0001), depicted in Figure S.22, in the case of high coverage, are similar to NH*: there is again only one energy well present that is associated with H abstraction.

**Figure 10:**
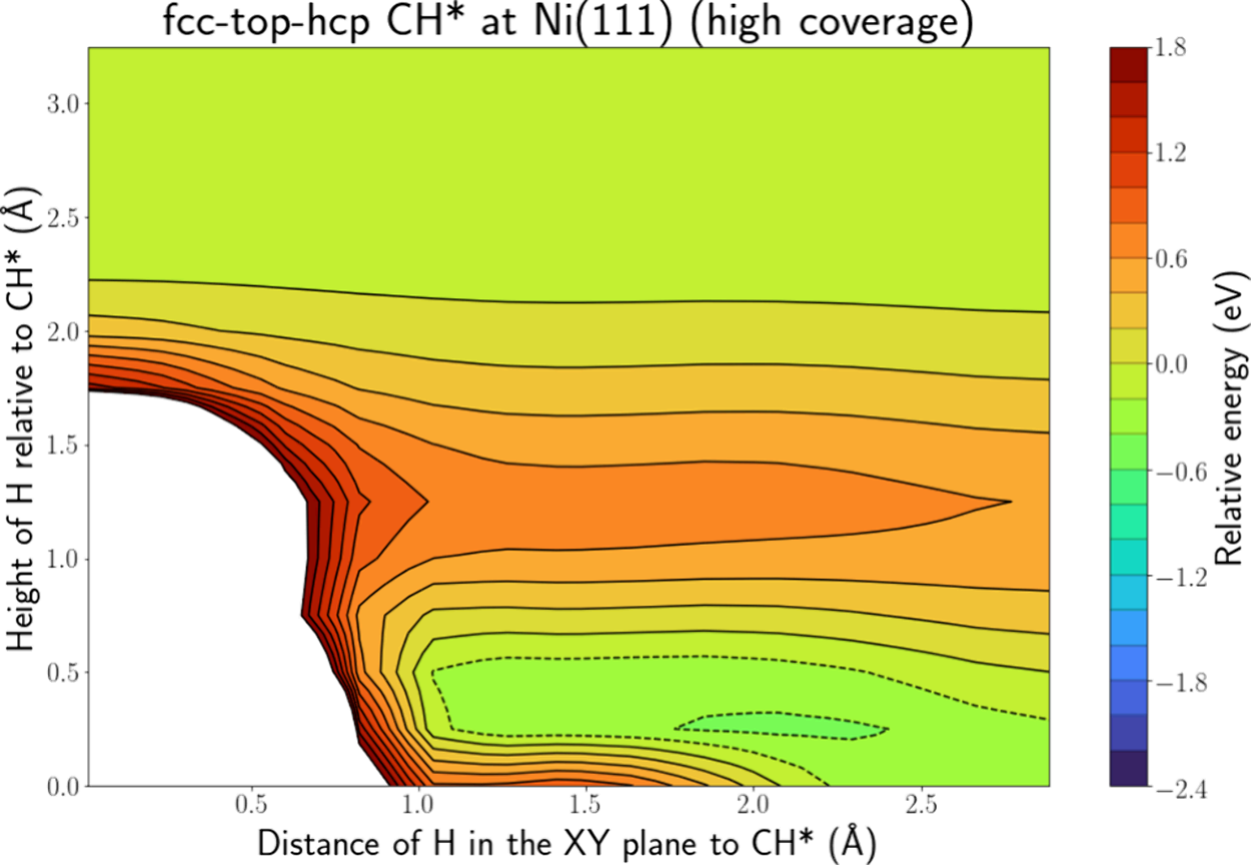
PES intersection for H(g) + CH* at the Ni(111) surface along the fcc-top-hcp line for a high coverage of CH*.

Finally, Figure 10 and Figure S.23 show the PES intersection for H(g) + CH* at Ni(111) along the fcc-top-hcp and fcc-bridge-hcp lines when all 9 fcc sites are occupied with CH*. In contrast with NH*, no energy well associated with H abstraction is present. This can be explained by the fact that the C–H bond is stronger than the N–H bond, and thus, H abstraction is more difficult. Furthermore, also in contrast with NH*, a shallow adsorption energy well is present. This well again extends toward the C atom, pointing to the possibility of an HA mechanism. However, to reach the energy well, especially the region closer to the C atom, the H radical has to overcome a barrier of roughly 0.8 eV. Hence, for H(g) + CH*, reflection or adsorption seem more probable than an ER or HA reaction.

In summary, for atomic adsorbates, we can conclude that the ER probability will increase with coverage, as the surface simply becomes harder to reach, although not all collisions with an adsorbate will lead to an ER reaction. For other adsorbates, the picture is less uniform. In the cases of NH* and NH_2_*, the ER reaction probability increases with coverage, but the product distribution will also be influenced. While in the case of CH*, adsorption and HA formation also become more difficult, there seems to be no increase in ER reactivity. While these conclusions might seem obvious, they are not always reflected in kinetic plasma catalysis models.

### 5.5 Perspective and Recommendation for Plasma-Catalytic Kinetic Models

From the literature discussed in Section 2 and our own results, there are obviously discrepancies between fundamental studies of ER reactions involving a H atom and how these reactions are modeled in plasma catalysis.

First, it is clear that some ER reactions found to be critically important in kinetic models are unlikely to play this vital role. For example, Du et al.^23^ stated that the ER reaction H(g) + CH_3_* will be responsible for CH_4_ production in plasma-catalytic CO_2_ hydrogenation. From both our own findings and the work of Zhou et al.,^24^ it is clear that this ER reaction is unlikely to happen, and when it occurs, it will not exclusively lead to CH_4_ formation but also to CH_2_* and H_2_ formation. The reason that some ER reactions are unlikely to happen is mostly due to steric hindrance caused by atoms that block the H atom from reaching the atom it needs to bind to. This is also illustrated by the fact that we find ER reactions to be more likely for atomic adsorbates.

Second, our results indicate that the HA mechanism and adsorption will become more important and the ER mechanism less important, the more sterical hindrance is present. The HA mechanism is typically not included in plasma-catalytic models. It is assumed to have no activation barrier, just like the ER mechanism, so one could argue that both ER and HA reactions can be modeled in the same way in a kinetic model. However, once the H atom is diffusing over the surface, as it does in the HA mechanism, it can react with all adsorbed species. This means that barrier-free reactions with all adsorbates should be included in the reaction set, which is not always the case in kinetic models. Furthermore, we found that HA reactions with atomic adsorbates can have a barrier.

Third, our results indicate that the surface coverage plays a vital role in determining the mechanism. At low coverage, i.e., as long as there is empty space on the surface near the adsorbate, ER reactions involving steric hindrance will likely not be important enough to significantly influence the reaction mechanism. Furthermore, the coverage can also play a role in determining the product, e.g., in the case of NH*, there is no indication for H abstraction for a single adsorbate, but at higher coverages, H abstraction is found to be even more likely than adsorption. This is also not included in kinetic models, as typically only one product of an ER reaction is taken into account.

Lastly, our results indicate that the metal surface may influence the ER probability. Overall, we conclude that kinetic models seem to overestimate the importance of ER reactions.

Hence, we make some recommendations for kinetic plasma catalysis models to avoid overestimating the importance of ER reactions:

- When studying ER reactions, it is crucial to include all possible ER reactions in the reaction set. For instance, when H(g) + CH* is included, the reactions of H(g) with all other adsorbates should also be included. Furthermore it is also necessary to include different products for the same ER reactions, even if these products do not contribute to the formation of a desired compound. This can be done by introducing branching ratios for the different products that might be dependent on the coverage.
- ER reactions with atomic adsorbates are found to be barrier-free; hence, we recommend that the rate is calculated based on a formula derived from collision theory containing a sticking coefficient, to account for the fact that the exothermicity of the ER reaction can lead to breaking of the formed bond.
- When the ER reaction rate coefficients are calculated using a 0 eV enthalpy barrier, it is important to introduce a sticking coefficient that accounts for the fact that not all adsorbates will be in a favorable conformation for an ER reaction at the moment of collision.
- The rate coefficients of the ER reactions should decrease when there is more sterical hindrance. For instance, NH* + H(g) should have a higher rate coefficient than NH_2_* + H(g). This can be done via the sticking coefficient discussed in the previous point. It is reasonable to assume that ER reactions with adsorbates that have the same level of sterical hindrance, e.g., atomic adsorbates, have a similar rate coefficient.
- It is important to include a coverage threshold for ER reactions with an adsorbate that has sterical hindrance, e.g., H(g) + CH*, and set their rate coefficient to zero when the coverage is below this threshold. This reflects the fact that, as long as there is free space on the surface next to an adsorbate with sterical hindrance, adsorption is far more likely.
- When a kinetic model predicts that an ER reaction is important, we recommend to vary the rate coefficient, i.e., to perform a sensitivity analysis, and to construct a PES profile, as we showed in this work, to evaluate how probable the ER reaction is.

It is obvious that more research is needed to quantify our recommendations and conclusions. For instance, we recommend using sticking coefficients, but the value of these coefficients is unclear. However, they might be obtained through MD simulations and molecular beam experiments. If these coefficients are determined as a function of initial incidence energy and direction, as well as the rovibrational state, obtaining reaction rates for usage in, e.g., microkinetic modeling is straightforward. One would need to integrate the probabilities over the translational, rotational, and vibrational distributions as follows:
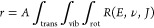
where *r* is the rate coefficient, *A* is the impact frequency factor, and *R* is the sticking coefficient dependent on the translation energy *E*, the vibrational state ν, and the rotational state *J*.

Also, we studied simple systems containing only one type of adsorbate, for the sake of simplicity, but in reality, the surface will be covered with different kinds of adsorbates. Currently, we know very little on how an HA on the surface will behave when multiple adsorbates are present. That is, will the HA just react with the closest adsorbate? The most obvious way to study this is by AIMD simulations, but this method is computationally costly for a large number of reactions. As a lot of different systems and variables would need to be sampled and studied (most importantly, the incident angle and energy of the incident species, coverage, and different combinations of adsorbates on the surface), an enormous number of trajectories would be needed, in addition to long time scales. This would not be feasible using AIMD, because of the computational cost. Hence, the forces for the MD simulations would need to be calculated in a computationally cheaper but still an accurate way. This could be done by training a neural network potential.^52^ Likewise, rare event sampling approaches can help with reducing the computational cost associated with the time scale.

## 6 Conclusions

Plasma catalysis is an emerging technology that could help stop the acceleration of climate change. However, a lot of questions about the underlying mechanisms remain. One of these questions is how important are ER reactions for the surface chemistry, next to LH reactions. Most plasma catalysis kinetic models predict that they will be important and sometimes even vital for the surface chemistry. However, very little is known about these ER reactions, and consequently a lot of assumptions are used when incorporating these reactions in kinetic models. In this work, we take a critical look at these assumptions, based on the construction of PES intersections, supported by fundamental studies from literature. To our knowledge, it is the first time that such an approach has been applied in the context of plasma catalysis. We focus here on ER reactions relevant for CO_2_ hydrogenation and NH_3_ synthesis, i.e., where the gas species is a H atom, as these are among the most commonly studied reactions in plasma catalysis.

Our results of the PES intersections are in agreement with AIMD findings in literature, as far as they were available. Hence, we recommend that researchers use this method to study ER reactions that are predicted to be important by plasma catalysis kinetic models, as our method is computationally cheap. We find that, for the reactions studied here, adsorption is more probable than a reaction via the HA mechanism, which in turn is more probable than a reaction via the ER mechanism. We also conclude that kinetic models of plasma-catalytic systems tend to overestimate the importance of ER reactions. Furthermore, the probability of an ER reaction decreases when there is more sterical hindrance. For atomic adsorbates, the ER probability increases with the coverage. For other adsorbates, the influence of the coverage depends on the adsorbate. The total reaction probability, including both the HA and ER mechanism, might even decrease with rising coverage. We also find that, as opposed to what is often assumed in kinetic models, the choice of a catalyst, i.e., stronger or weaker binding metals, may also influence the ER reaction probability. Most of these findings may seem evident but are often not reflected in the way ER reactions are modeled for plasma catalysis.

Based on these findings, we make a number of recommendations on how to incorporate ER reactions in kinetic models, most importantly: (i) the inclusion of a sticking coefficient when the ER reaction is (assumed to be) barrier-free, to account for the difficult dissipation of reaction energy, (ii) the reaction rate coefficient should become lower the more sterically hindered the adsorbate is, and (iii) multiple products, of which the distribution can depend on the coverage, should be taken into account for one ER reaction. To further elucidate the role of ER reactions in plasma catalysis, MD simulations are needed, and we recommend the use of neural network potentials to keep the computational cost under control.

## Supporting Information Available

Convergence of computational parameters (S.1); PES intersection figures (S.2); and C adsorption energy (S.3) (PDF)
