# Supplementary material for: Plasma Catalysis Modeling: How Ideal Is Atomic Hydrogen for Eley–Rideal?
Source: J Phys Chem C Nanomater Interfaces. 2024 Jul 1;128(27):11196–209. doi: 10.1021/acs.jpcc.4c02193 (PMC11247482; doi:10.1021/acs.jpcc.4c02193)
Supplement: Supplementary file 1 — jp4c02193_si_001.pdf [file jp4c02193_si_001.pdf]

Plasma Catalysis Modelling: How Ideal is Atomic Hydrogen for Eley-Rideal?

Roel Michiels[a], Nick Gerrits[a,b,*], Erik Neyts[a] and Annemie Bogaerts[a,+]

[a] Research group PLASMANT, Department of Chemistry, University of Antwerp, Universiteitsplein 1, BE-2610 Wilrijk-Antwerp, Belgium

[b] Leiden Institute of Chemistry, Gorlaeus Laboratories, Leiden University, P.O. Box 9502, 2300 RA Leiden, The Netherlands

*: n.gerrits@lic.leidenuniv.nl, +31715271328

+: annemie.bogaerts@uantwerpen.be, +3232652377

S.1 Convergence of computational parameters for DFT calculations

The adsorption energy of species, E_{ads}, is defined as:

$$E_{ads} = E_{adsorbate+surface} - (E_{surface} + E_{adsorbate})$$

Where $E_{adsorbate+surface}$, $E_{surface}$, and $E_{adsorbate}$ are the total energies of the adsorbate on the slab, the clean slab and the gaseous adsorbate, respectively.

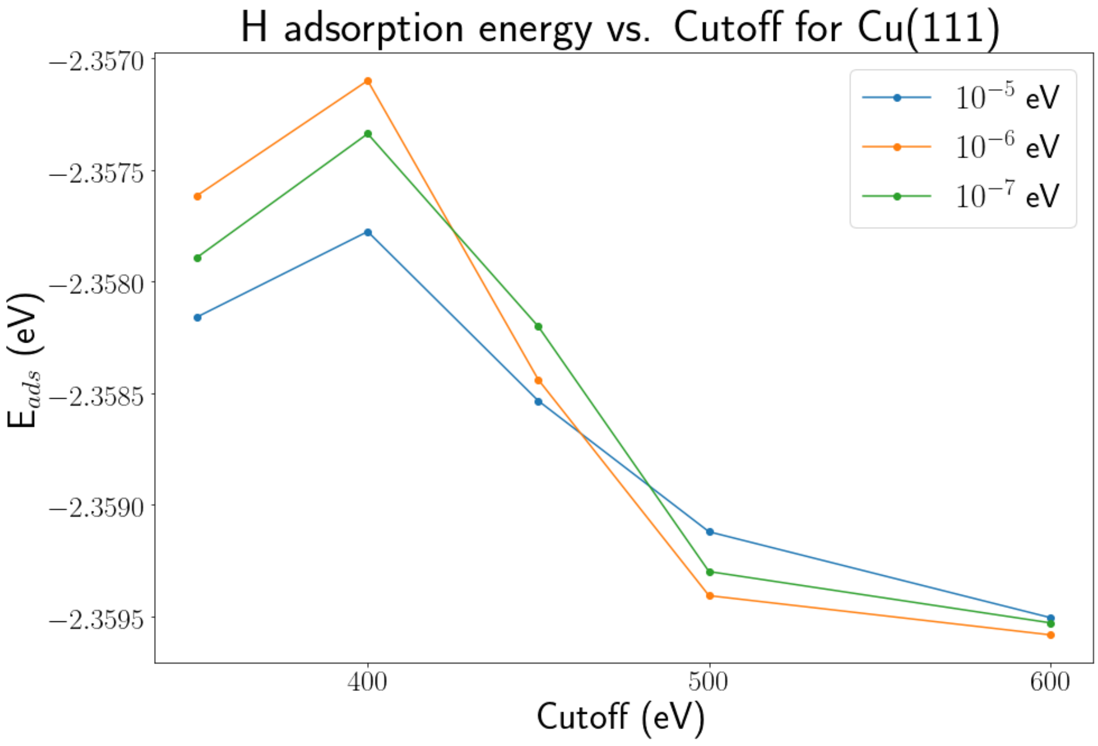

Figure S1: Convergence of the H adsorption energy on Cu(111) as a function of the cutoff. The different lines show the convergence for different electronic SCF convergence criteria.

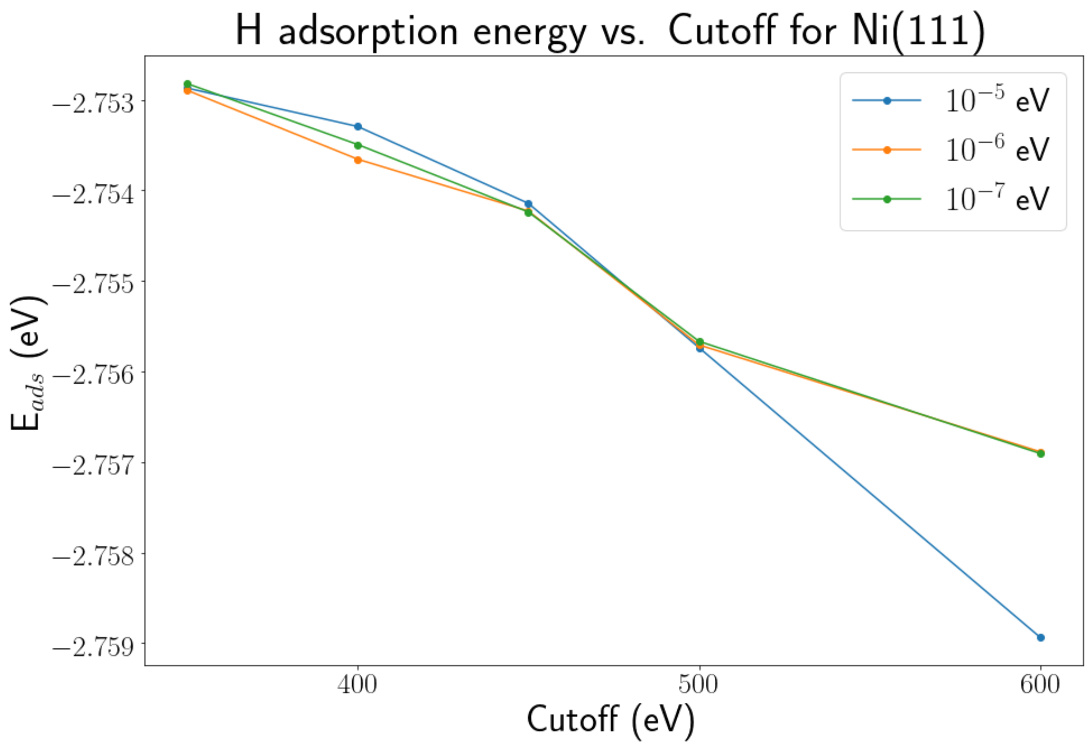

Figure S2: Convergence of the H adsorption energy on Ni(111) as a function of the cutoff. The different lines show the convergence for different electronic SCF convergence criteria.

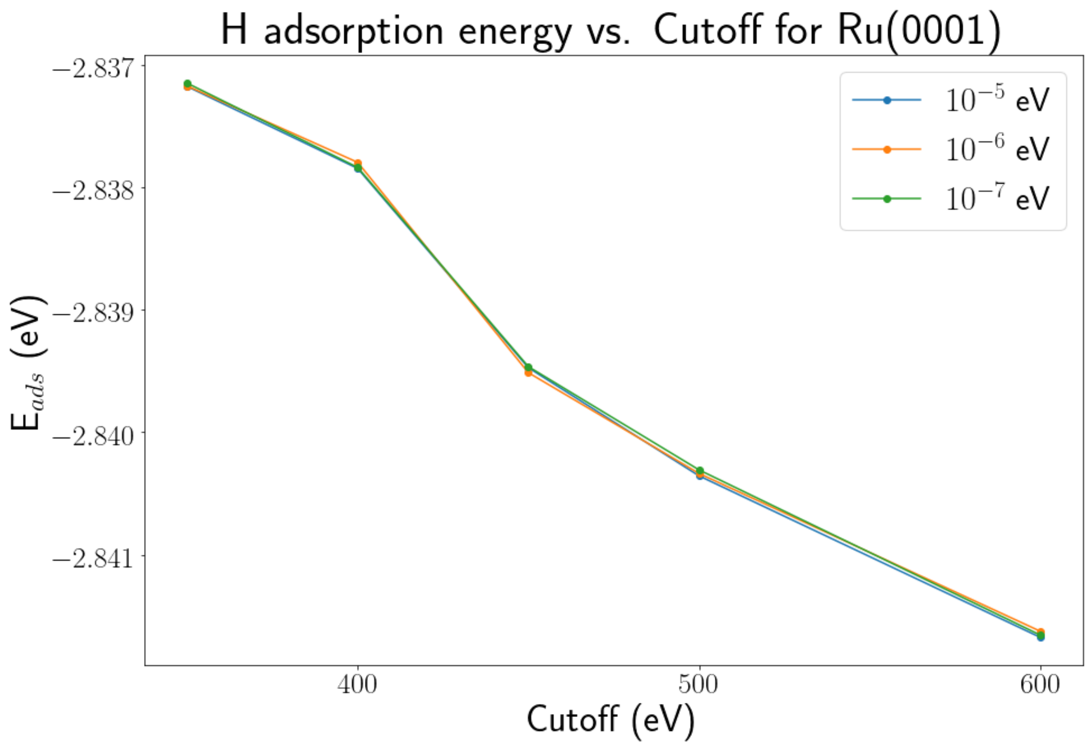

Figure S3: Convergence of the H adsorption energy on Ru(0001) as a function of the cutoff. The different lines show the convergence for different electronic SCF convergence criteria.

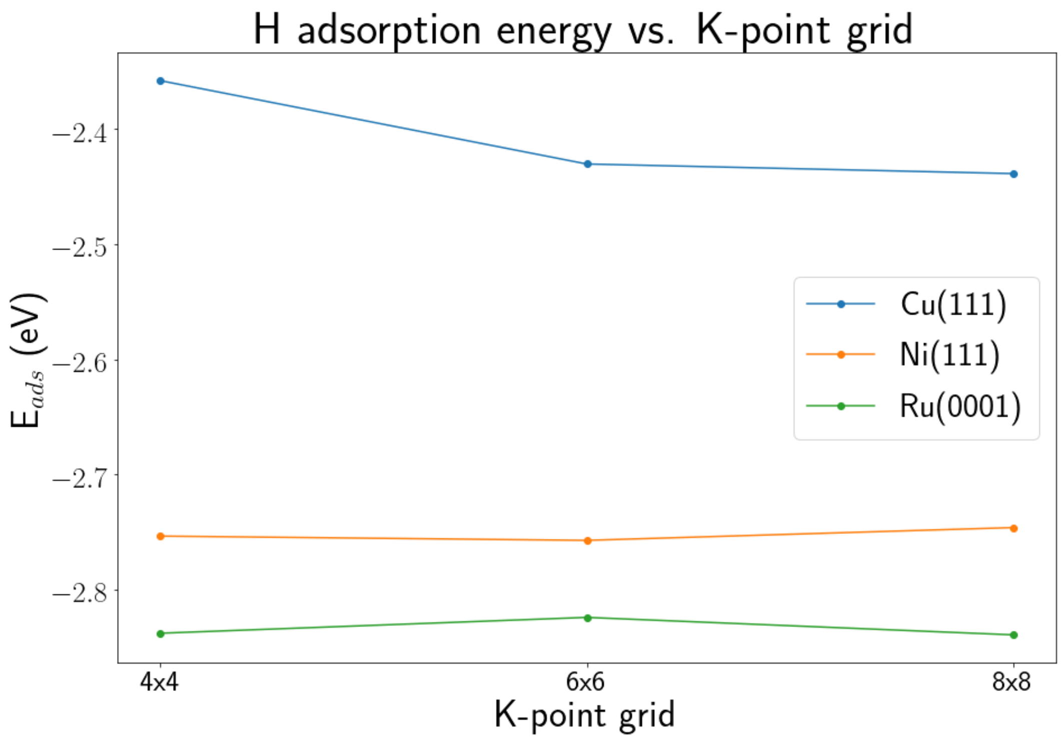

Figure S4: Convergence of the H adsorption energy as a function of the K-point grid. The different lines show the convergence for different metals.

S.2 PES intersections

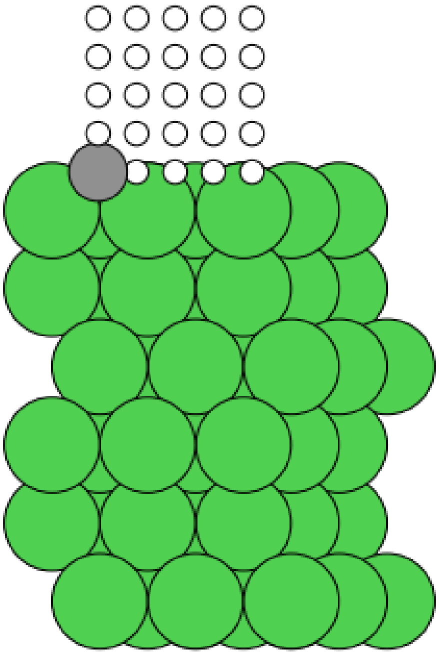

Figure S.5: Illustrative picture on how the PES intersections are constructed for H(g) + C* at Ni(111). The distance between the H atom positions is not to scale. The different positions of the H atom are represented by white spheres.

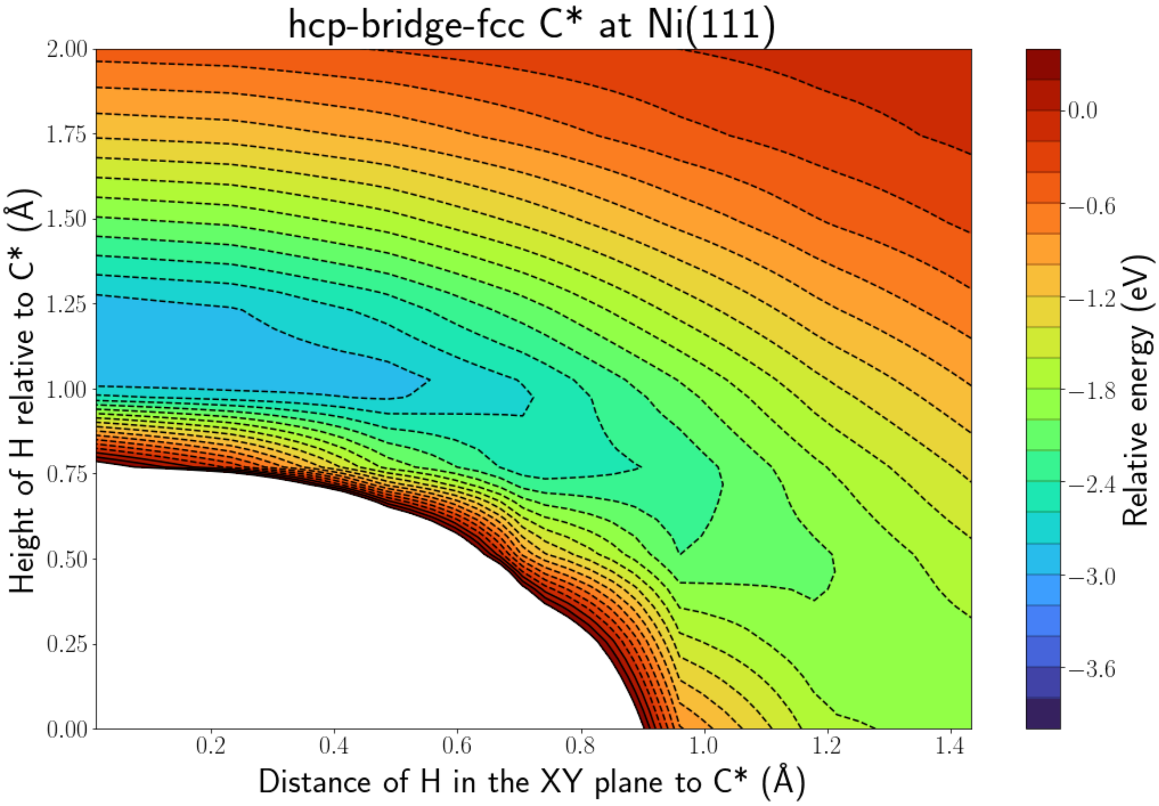

Figure S.6: PES intersection for H(g) + C* at Ni(111) surface along the hcp-bridge fcc line.

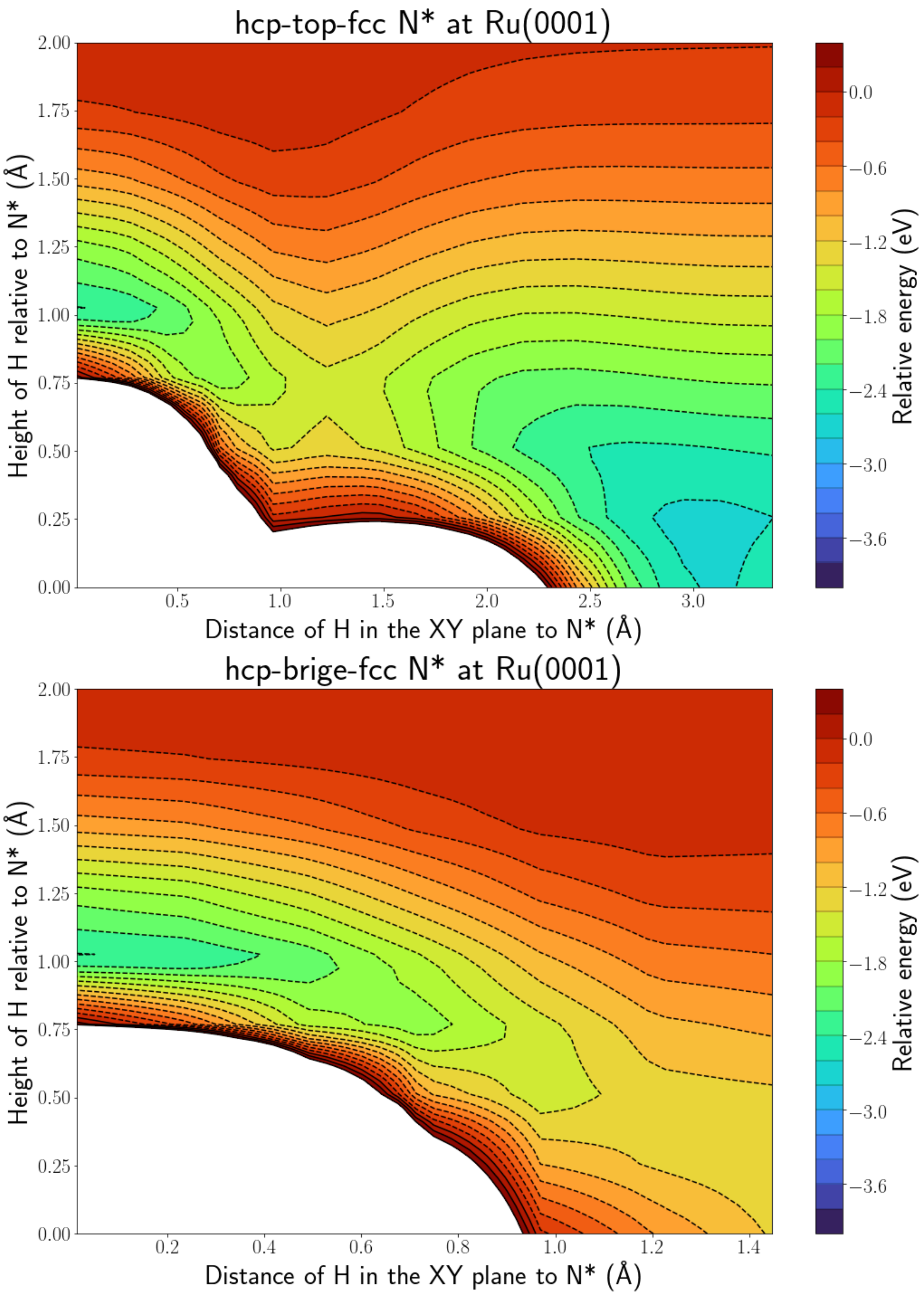

Figure S.7: PES intersection for H(g) + N* at Ru(0001) surface along the hcp-top-fcc line (top panel) and the hcp-bridge-fcc line (bottom panel).

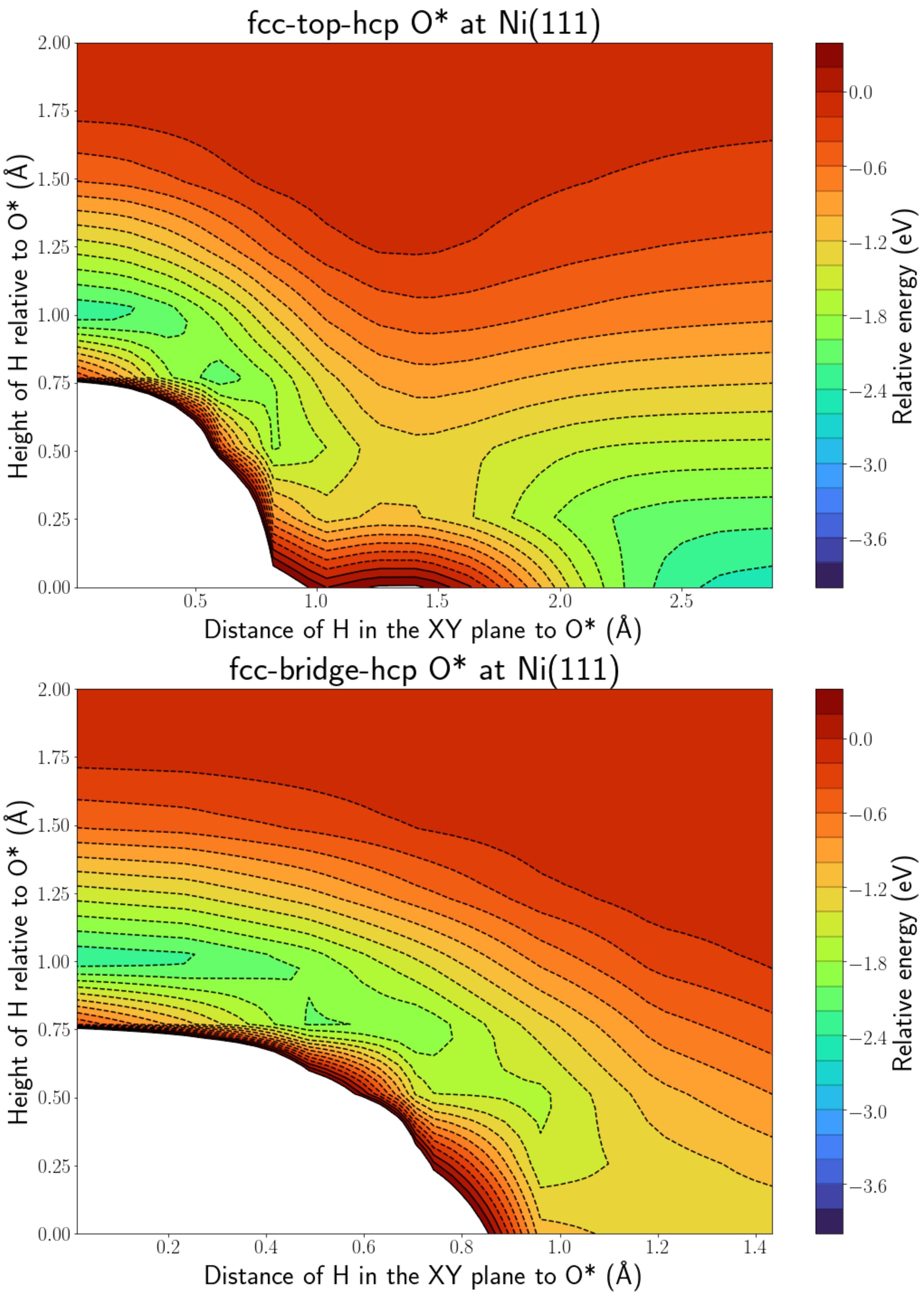

Figure S.8: PES intersection for H(g) + O* at Ni(111) surface along the fcc-top-hcp line (top panel) and the fcc-bridge-hcp line (bottom panel).

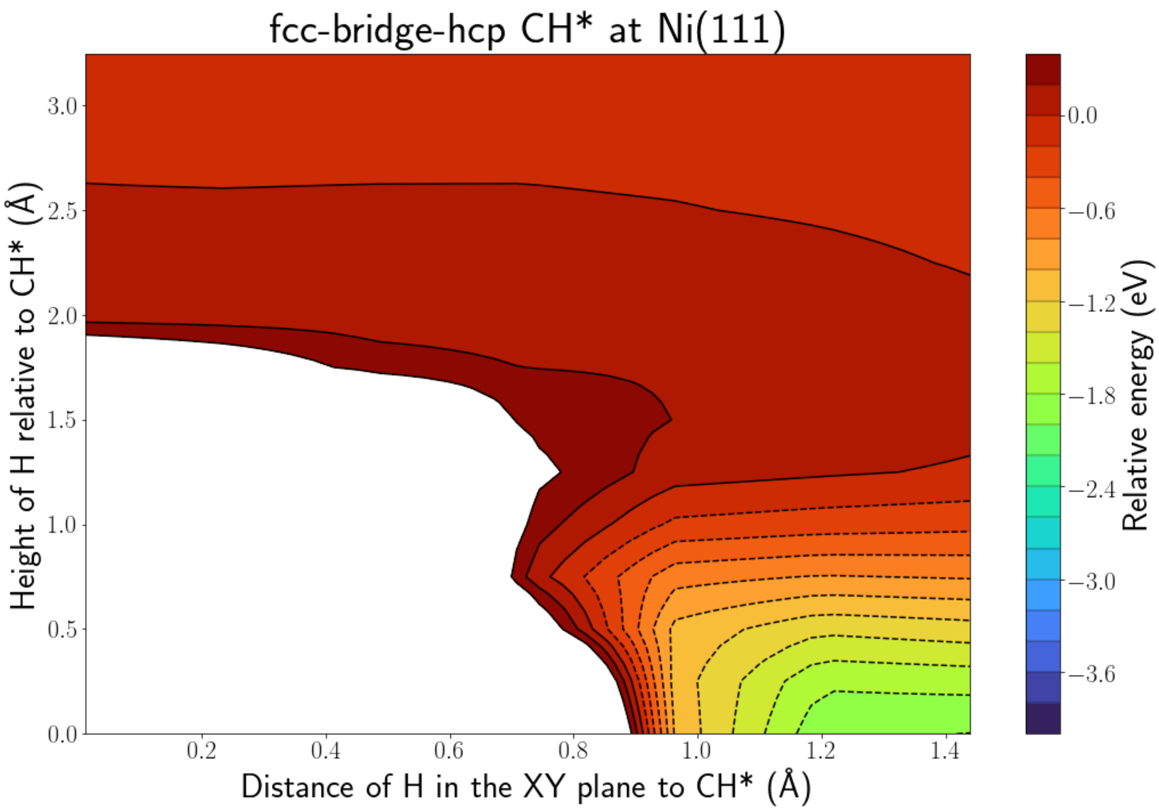

Figure S.9: PES intersection for H(g) + CH* at Ni(111) surface along the fcc-bridge-hcp line.

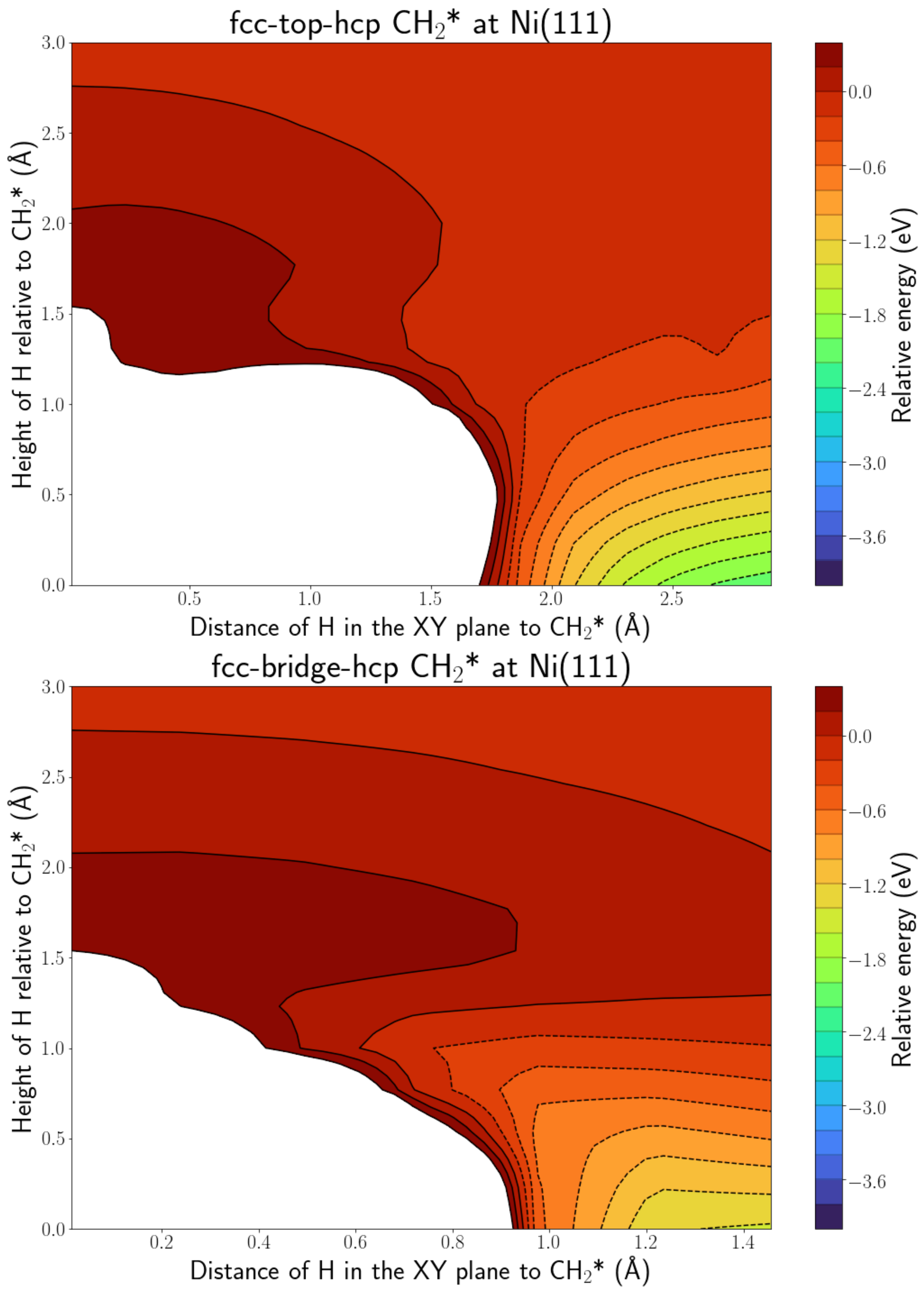

Figure S.10: PES intersection for H(g) + CH_2* at Ni(111) surface along the fcc-top-hcp line (top panel) and fcc-bridge-hcp (bottom panel).

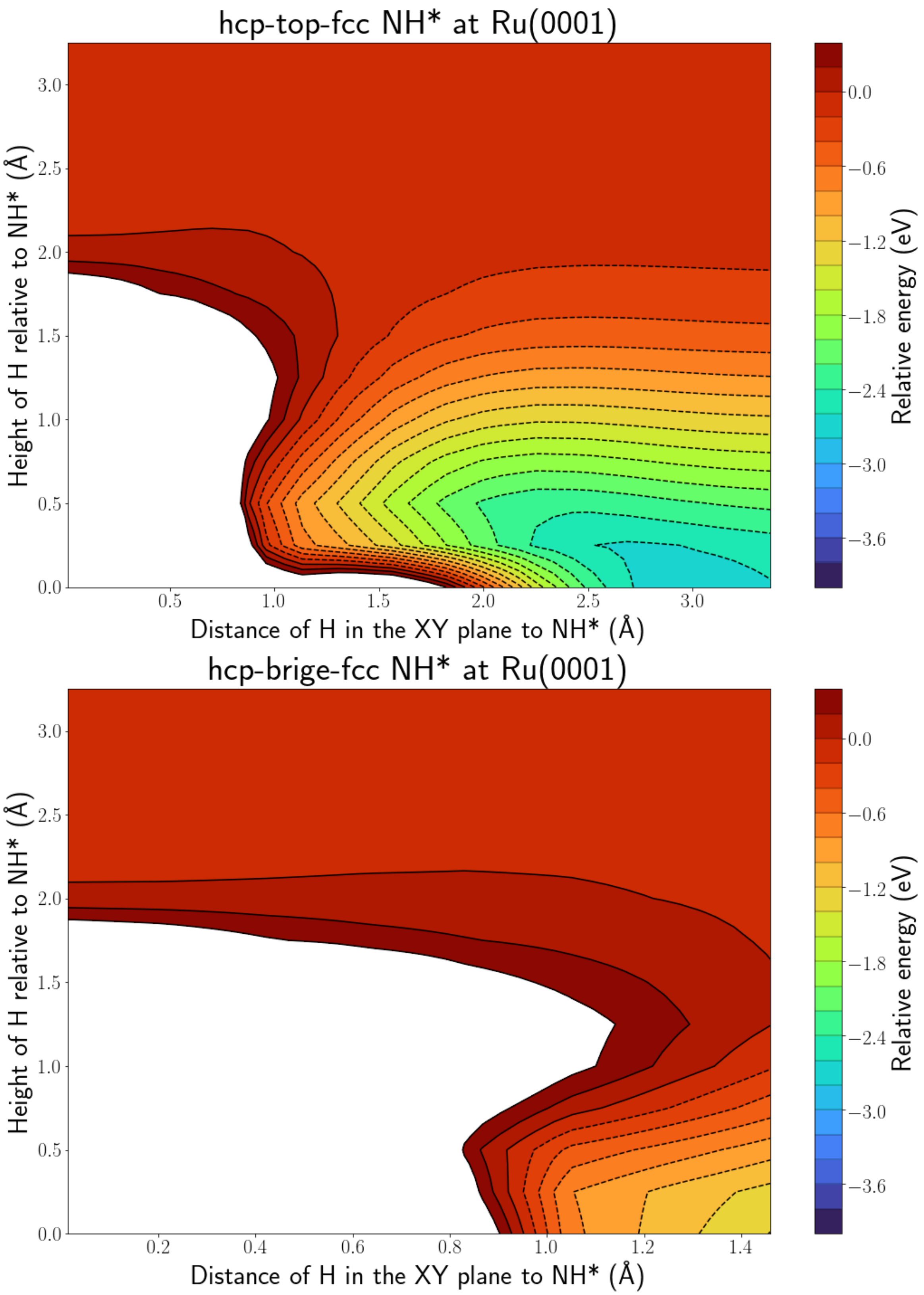

Figure S.11: PES intersection for H(g) + NH* at Ru(0001) surface along the hcp-top-fcc line (top panel) and the hcp-bridge-fcc line (bottom panel).

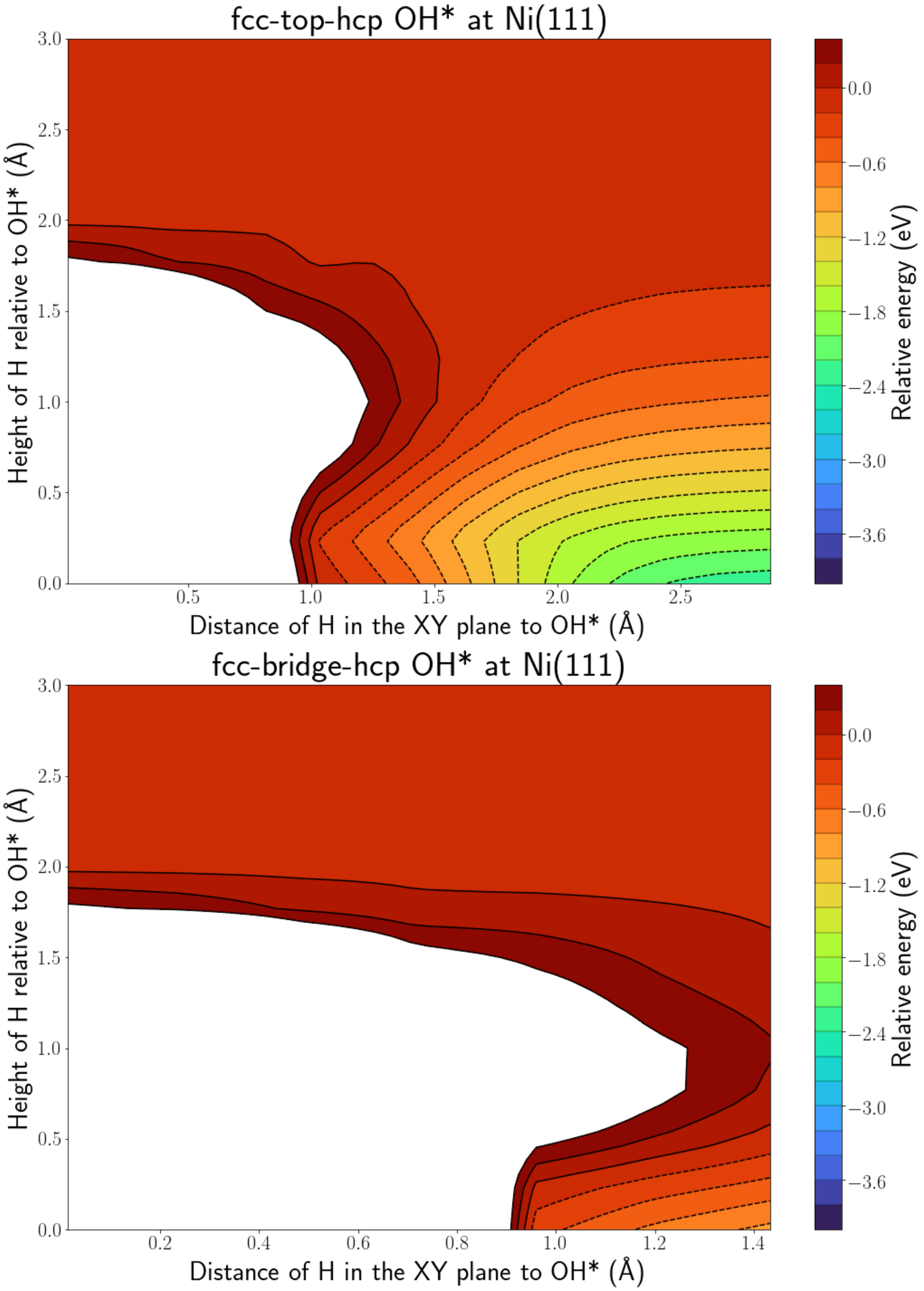

Figure S.12: PES intersection for H(g) + OH* at Ni(111) surface along the fcc-top-hcp line (top panel) and the fcc-bridge-hcp line (bottom panel).

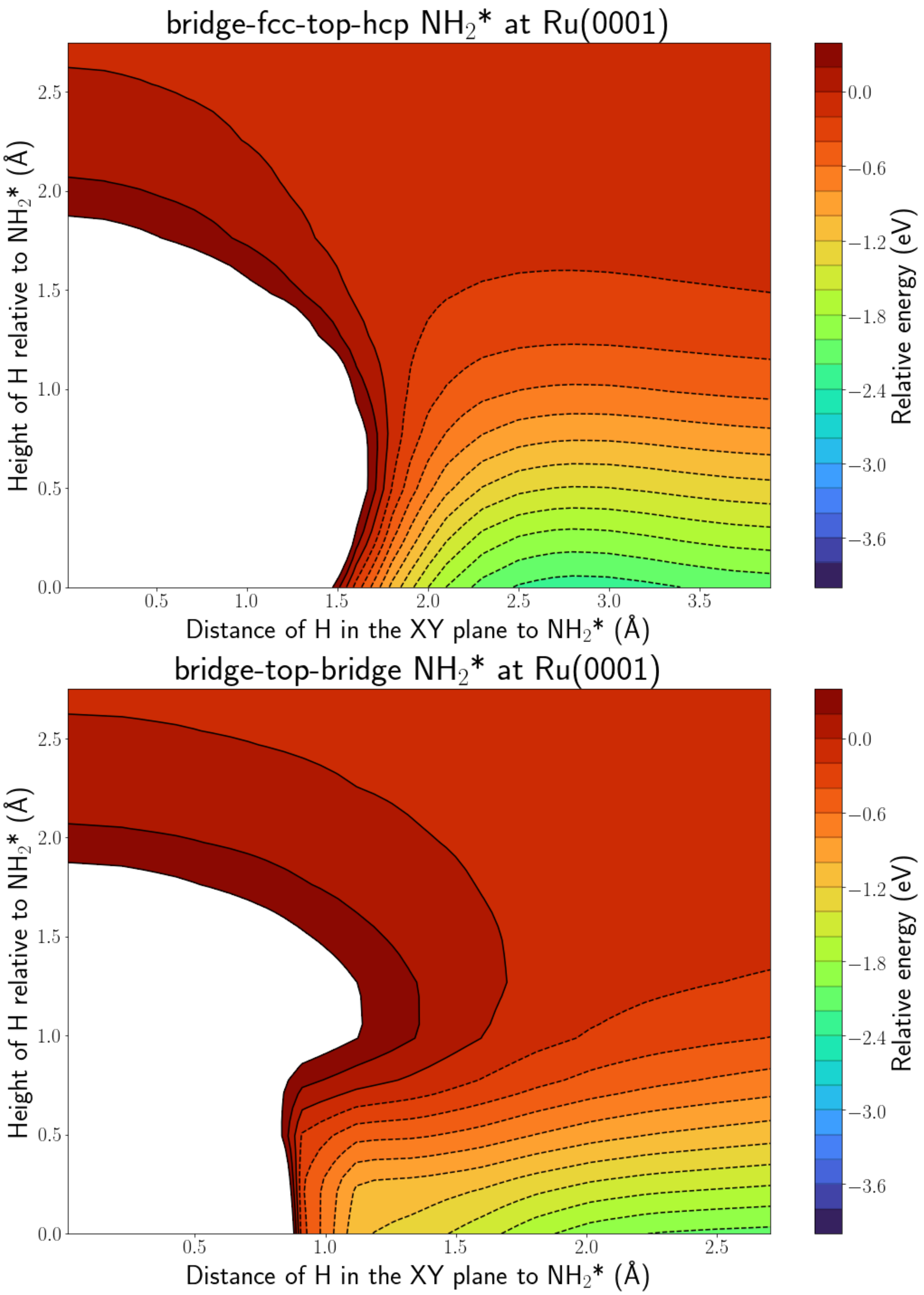

Figure S.13: PES intersection for H(g) + NH_2* at Ru(0001) surface along the bridge-fcc-top-hcp line (top panel) and the bridge-top-bridge line (bottom panel).

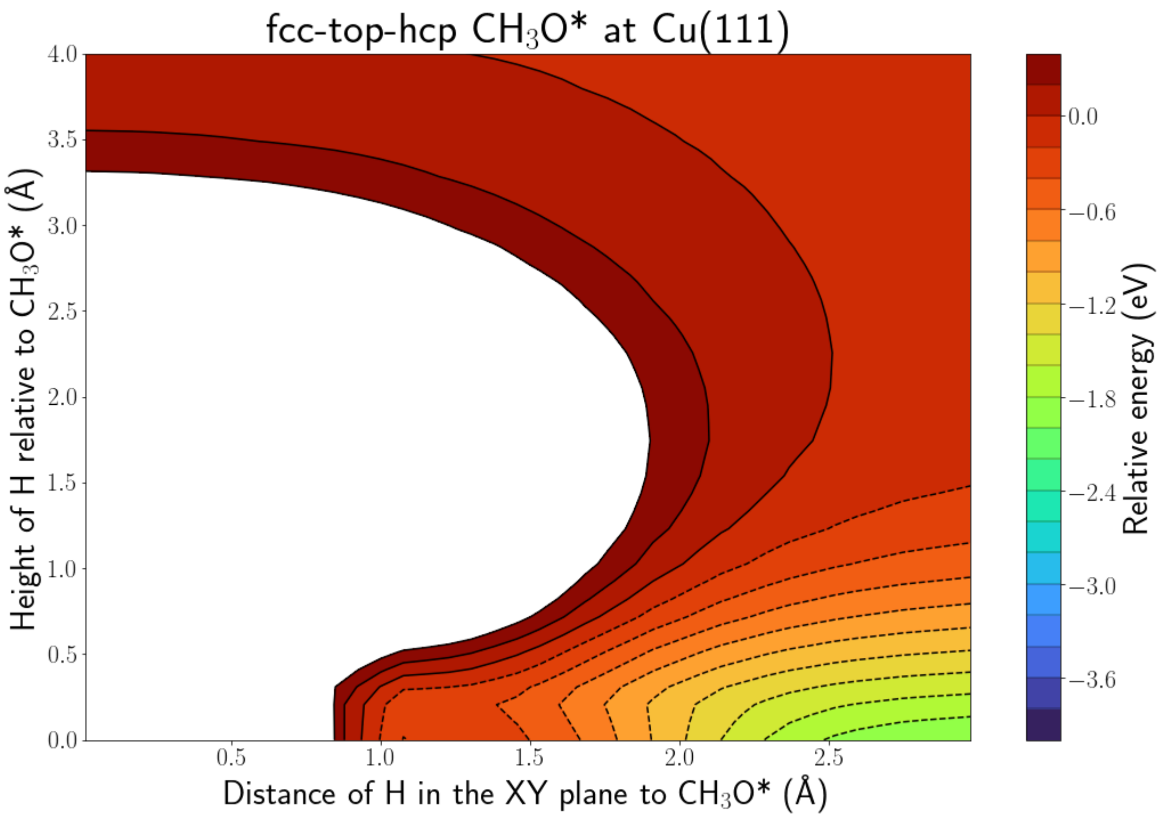

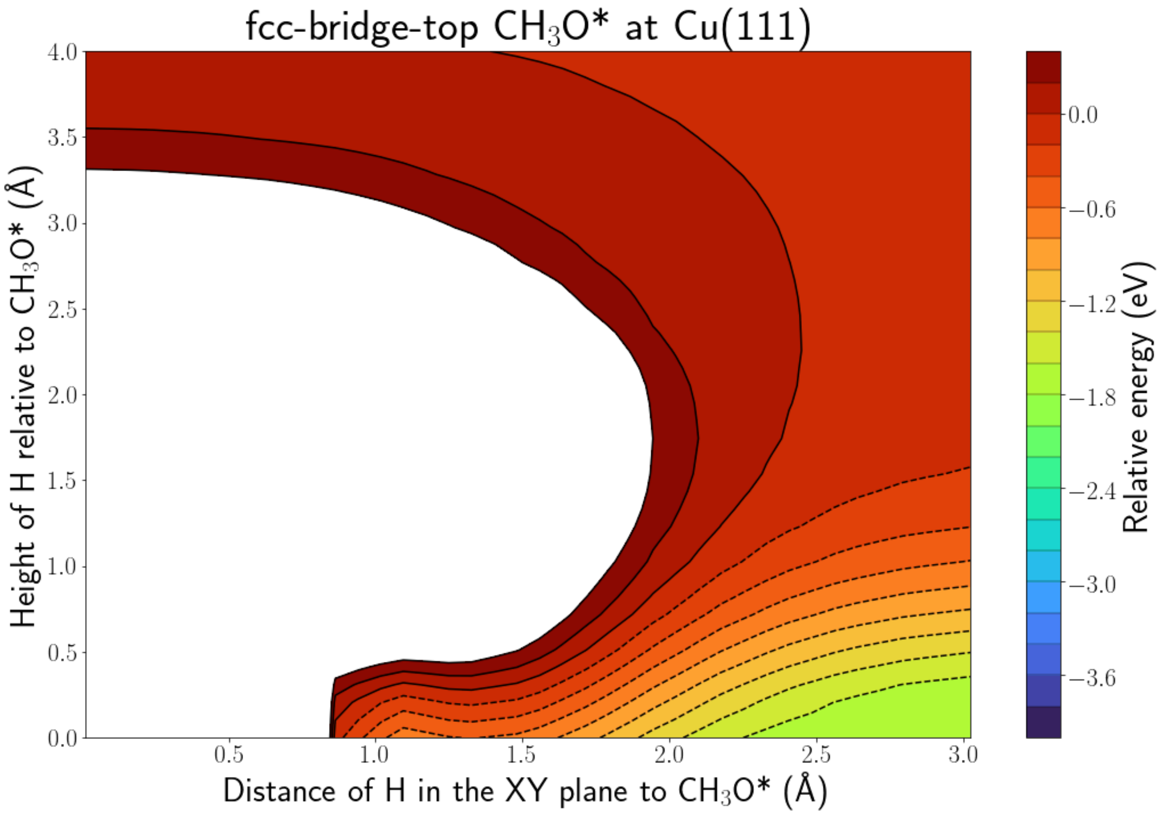

Figure S.14: PES intersection for H(g) + CH_3O* at Cu(111) surface along the fcc-top-hcp line (top panel) and the fcc-bridge-top line (bottom panel).

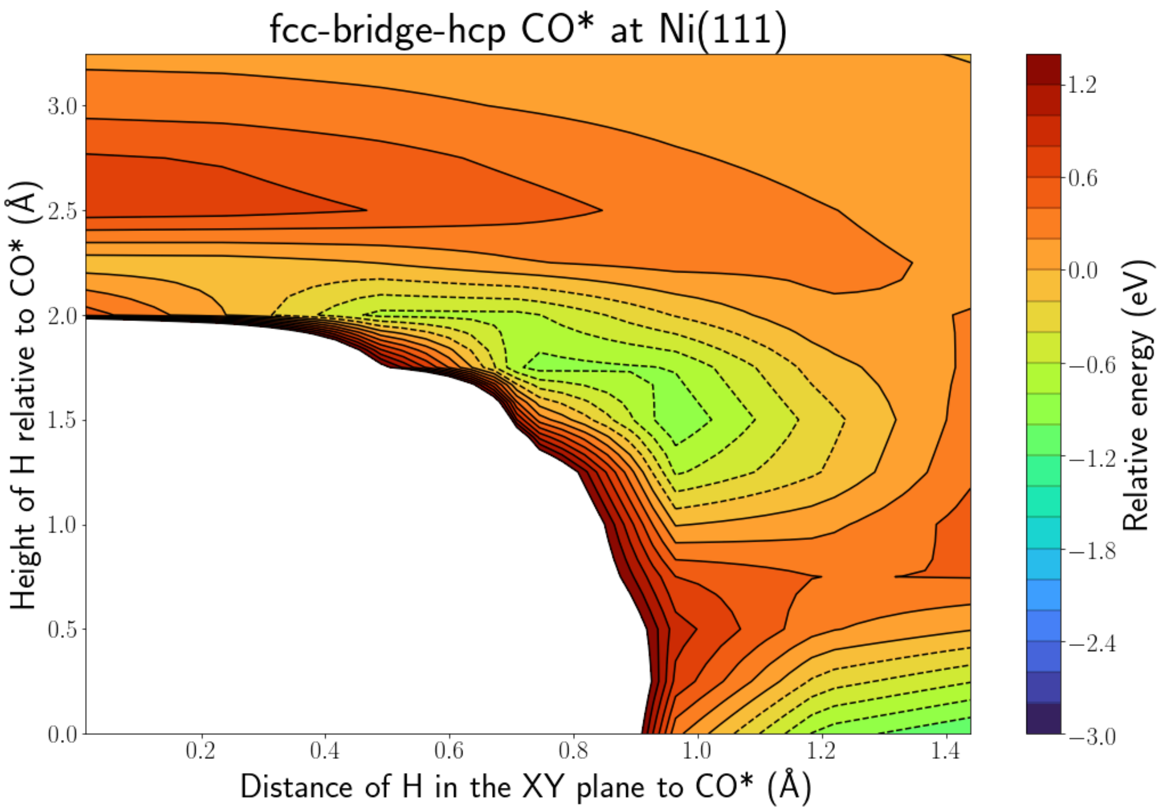

Figure S.15: PES intersection for H(g) + CO* at Ni(111) surface along the fcc-bridge-hcp line.

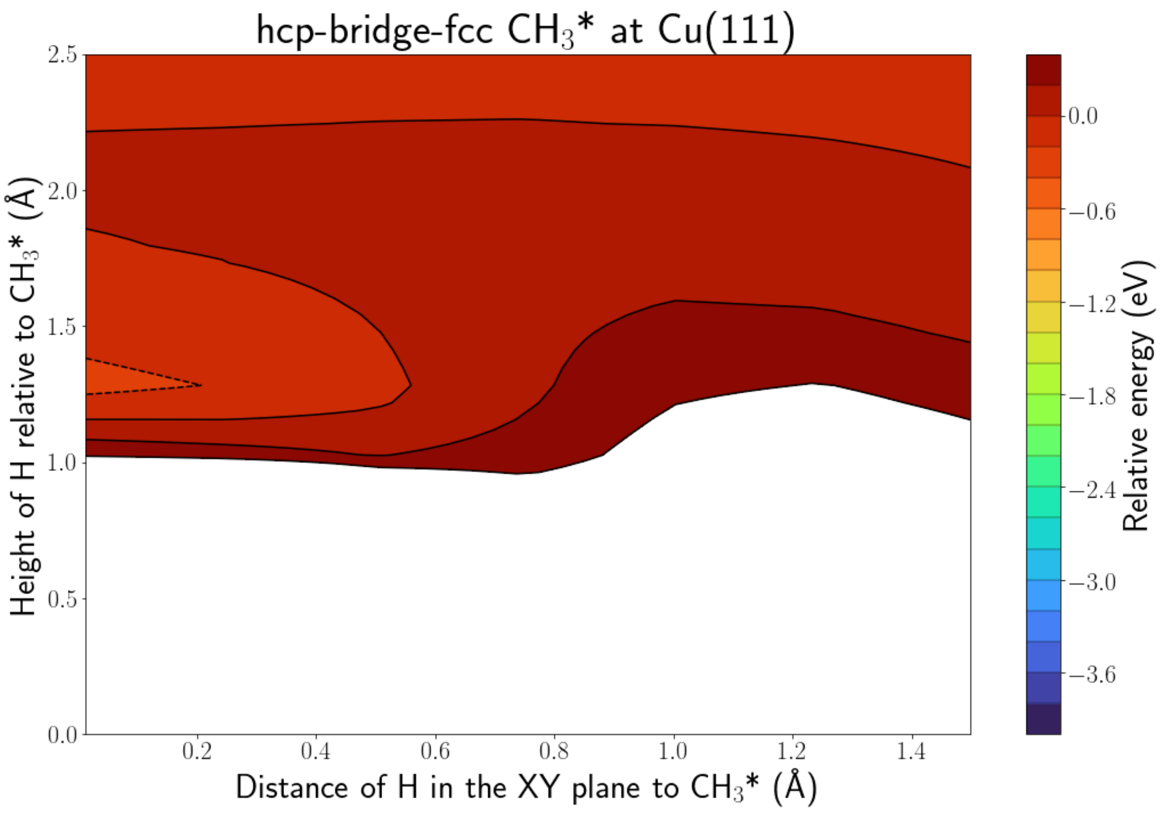

Figure S.16: PES intersection for H(g) + CH_3* at Cu(111) surface along the hcp-bridge-fcc line.

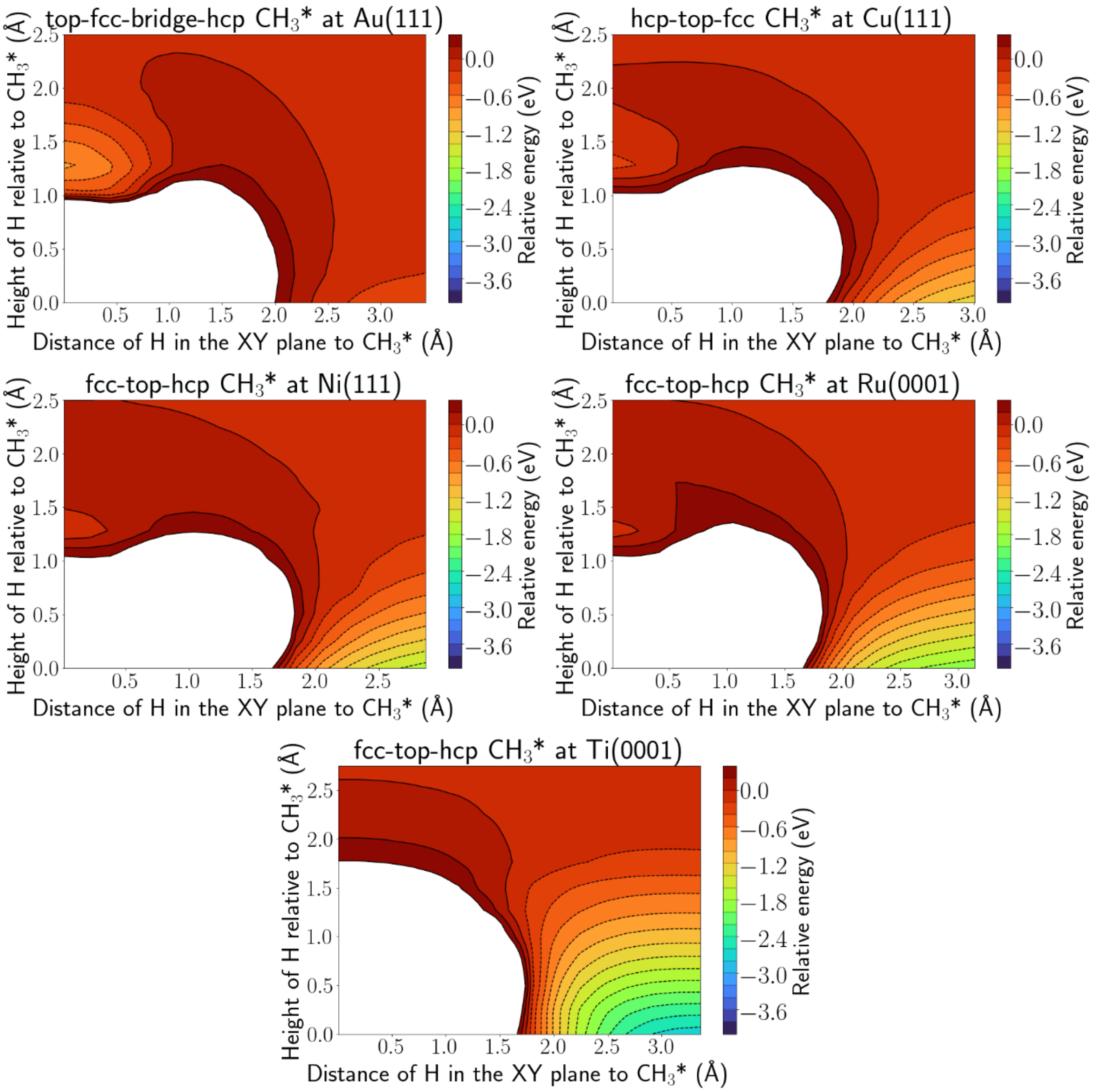

Figure S.17: PES intersection for H(g) + CH_3* at Au(111) (top left), Cu(111) (top right), Ni(111) (middle left), Ru(0001) (middle right) and Ti(0001) (bottom panel).

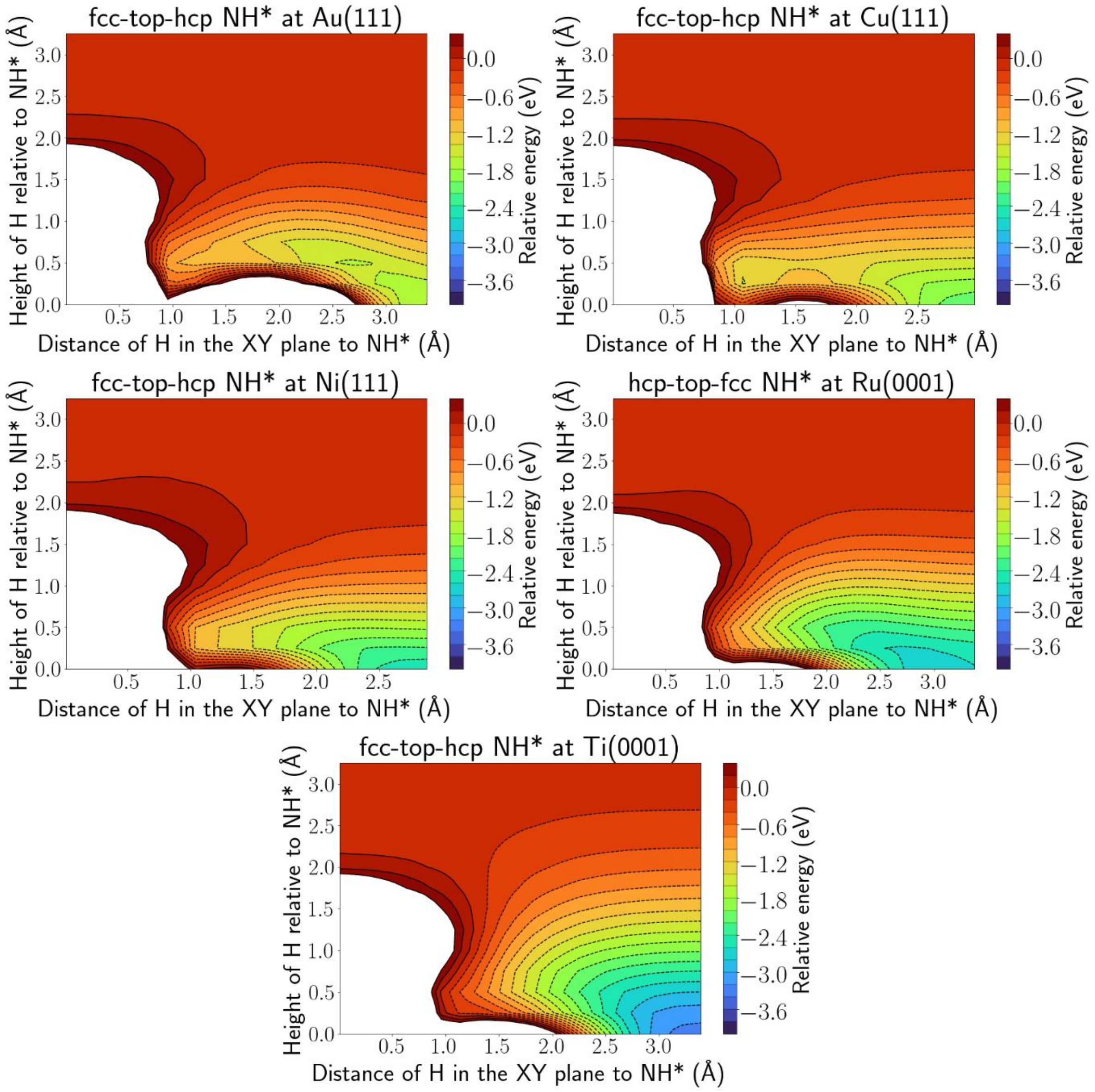

Figure S.18: PES intersection for H(g) + NH* at Au(111) (top left), Cu(111) (top right), Ni(111) (middle left), Ru(0001) (middle right) and Ti(0001) (bottom panel).

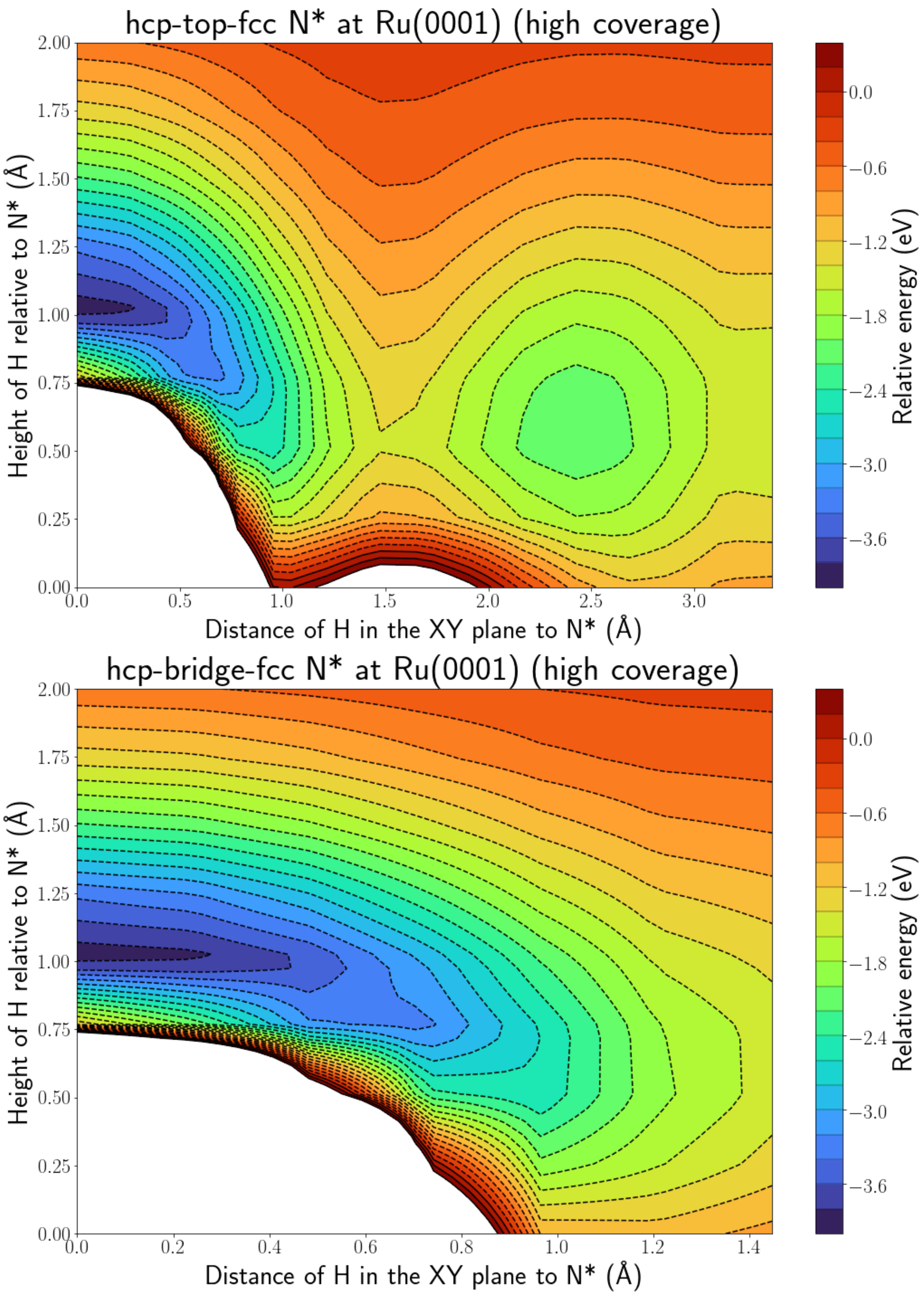

Figure S.19: PES intersection for H(g) + N* at Ru(0001) surface along the hcp-top-fcc line (top panel) and hcp-bridge-fcc line (bottom panel) for a high coverage of N.

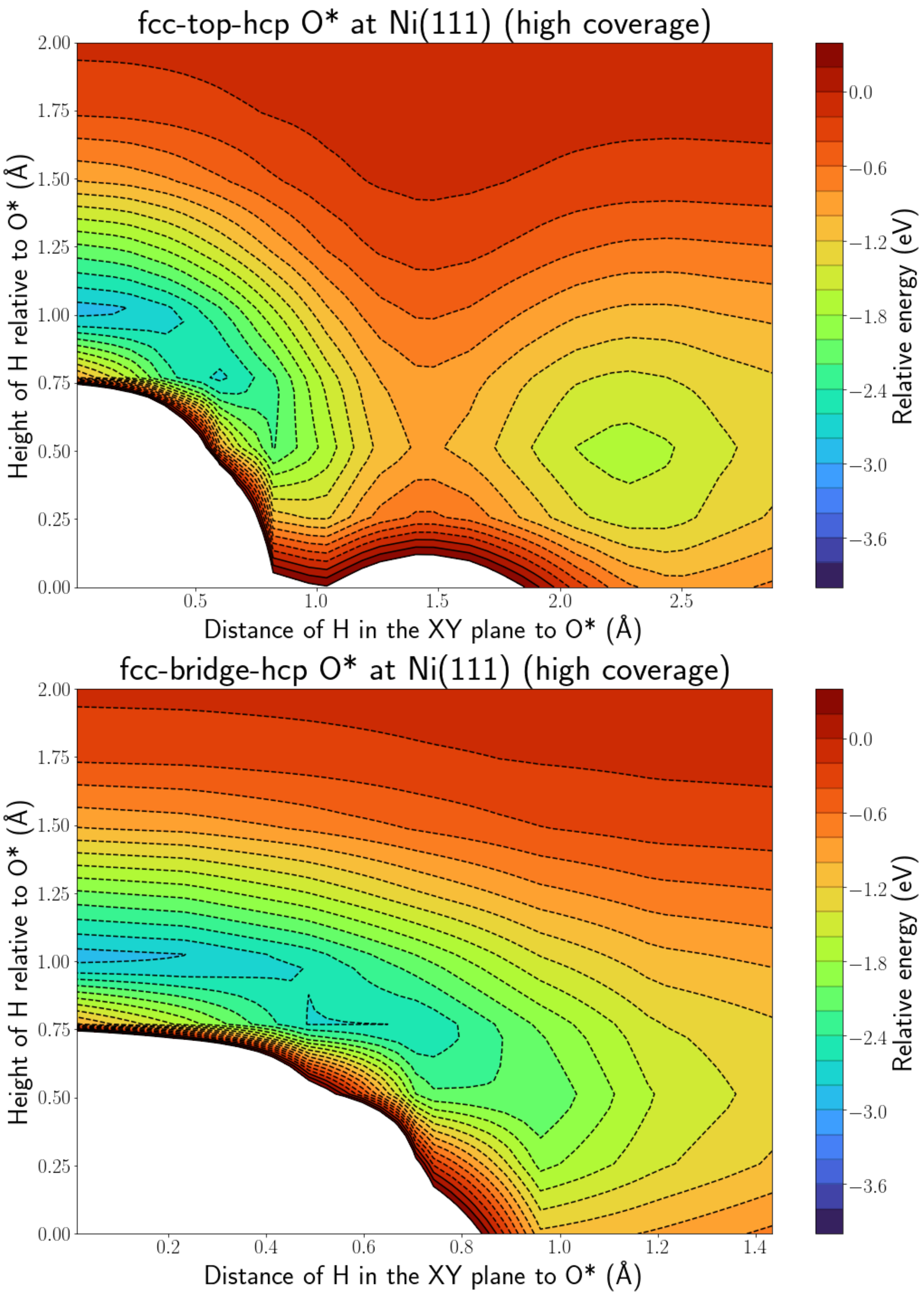

Figure S.20: PES intersection for H(g) + O* at Ni(111) surface for high O* coverage along the fcc-top-hcp line (top panel) and the fcc-bridge-hcp line (bottom panel).

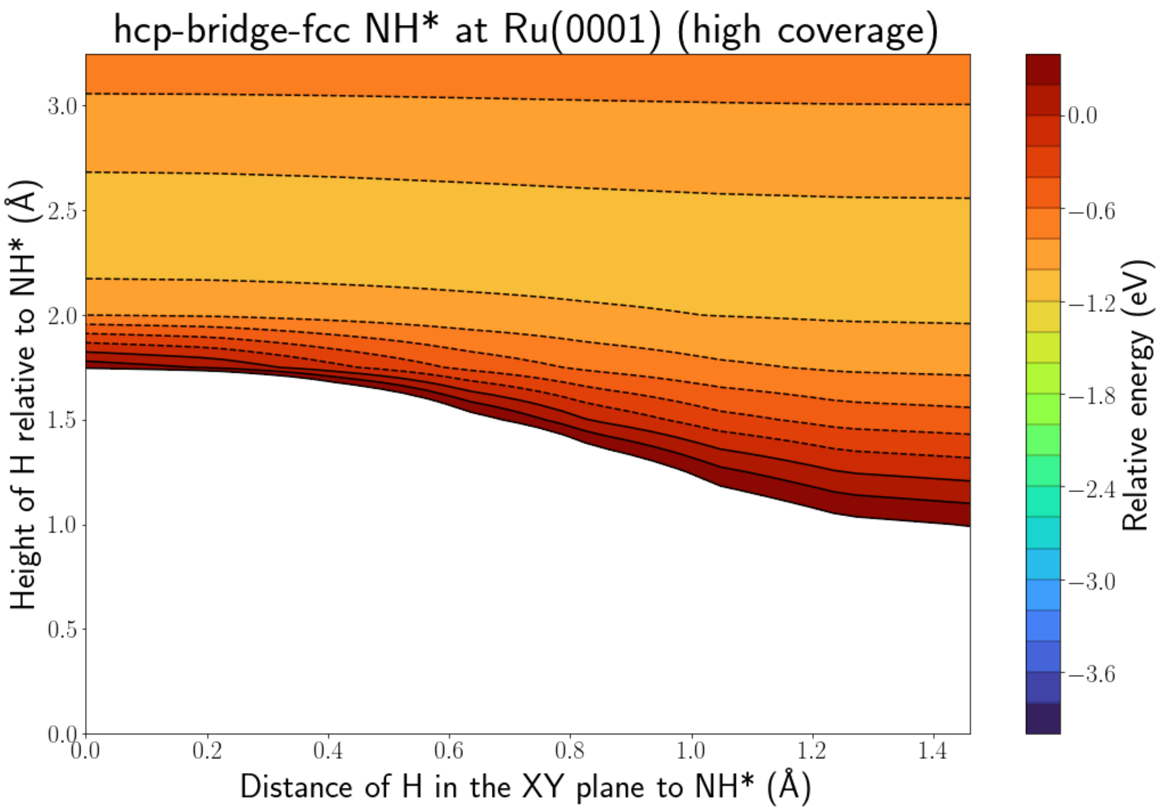

Figure S.21: PES intersection for H(g) + NH* at Ru(0001) surface along the hcp-bridge-fcc line for a high coverage of NH*.

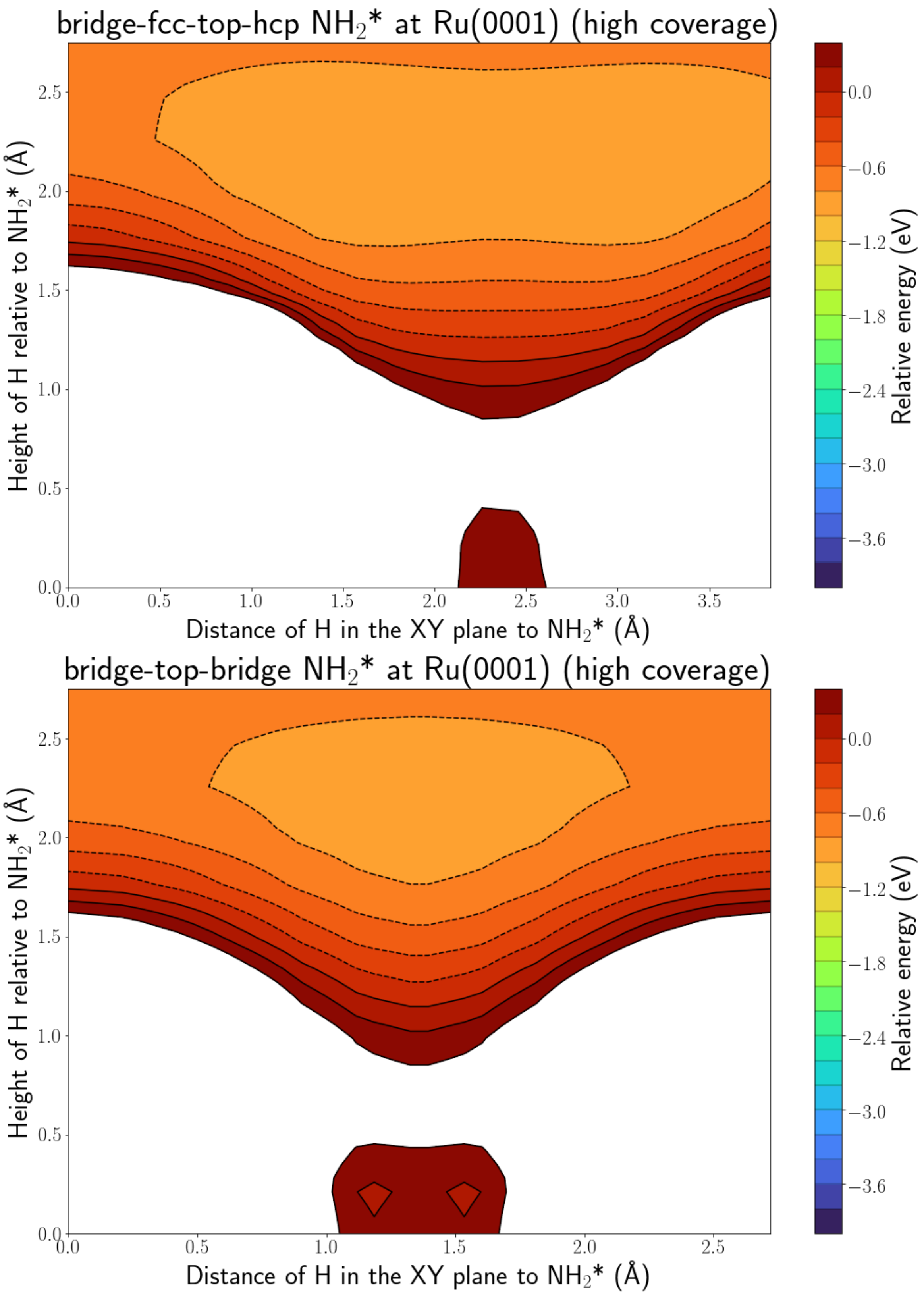

Figure S.22: PES intersection for H(g) + NH_2* at Ru(0001) surface along the bridge-fcc-top-hcp line (top panel) and the bridge-top-bridge line (bottom panel).

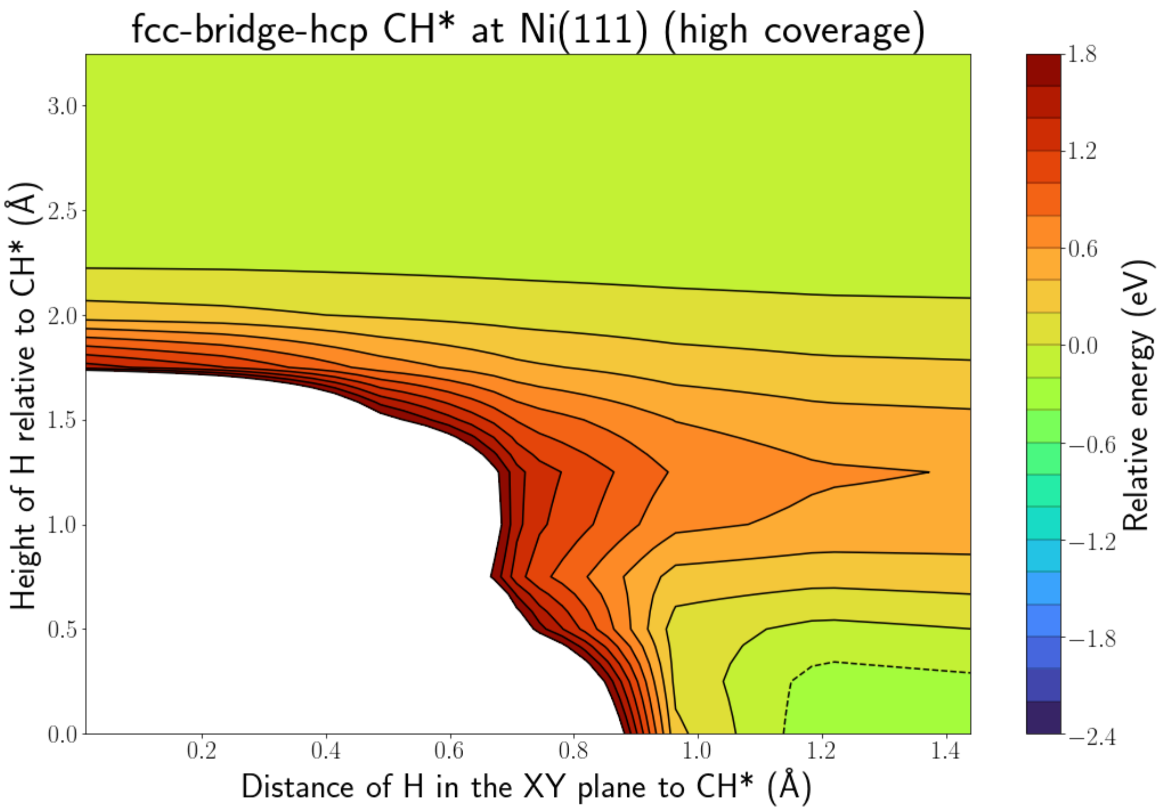

Figure S.23: PES intersection for H(g) + CH* at Ni(111) surface along the fcc-bridge-hcp line for a high coverage of CH*.

S.3 C adsorption energy

Table S.1: C adsorption energies in eV for the different metal surfaces.

Metal surface	Adsorption energy C atom (eV)
Au(111)	-3.82
Cu(111)	-4.23
Ni(111)	-6.21
Ru(0001)	-7.20
Ti(0001)	-7.56
